# Biological functions and emerging interplay of FGF21 and GDF15 in metabolic diseases and aging

**DOI:** 10.1186/s43556-026-00627-w

**Published:** 2026-10-30

**Authors:** Laura Salmón-Gómez, Susanne Keipert, Victoria Catalán, Amaia Rodríguez, Sara Becerril, Gema Frühbeck, Javier Gómez-Ambrosi

**Affiliations:** 1 Metabolic Research Laboratory, Clínica Universidad de Navarra, Irunlarrea 1, Pamplona, 31008 Spain; 2 Centro de Investigación Biomédica en Red-Fisiopatología de La Obesidad y Nutrición (CIBEROBN), Instituto de Salud Carlos III, Pamplona, Spain; 3 Obesity and Adipobiology Group, Instituto de Investigación Sanitaria de Navarra (IdiSNA), Pamplona, Spain; 4 Institute for Nutrition & Health, University of Navarra, Pamplona, Spain; 5 Department of Molecular Biosciences, The Wenner-Gren Institute, Stockholm University, Stockholm, Sweden; 6 Center of Brain Behavior and Metabolism (CBBM), Institute for Human Genetics, University of Lübeck, Lübeck, Germany; 7 Department of Endocrinology & Nutrition, Clínica Universidad de Navarra, Pamplona, Spain

**Keywords:** FGF21, GDF15, Obesity, Type 2 diabetes, MASLD, Aging

## Abstract

Metabolic diseases and age-related disorders represent a major global health burden driven by the increasing prevalence of obesity and obesity-associated complications. These disorders are characterized by chronic disturbances in energy homeostasis. A key feature linking metabolic disease and aging is the activation of stress-related pathways, which contribute to progressive metabolic dysfunction. Among the endocrine mediators of cellular stress, fibroblast growth factor 21 (FGF21) and growth differentiation factor 15 (GDF15) have emerged as key regulators of metabolic homeostasis. Both are induced by similar stress conditions, including nutrient imbalance, inflammation, oxidative and endoplasmic reticulum (ER) stress and mitochondrial dysfunction, and are elevated in pathophysiological conditions such as obesity, type 2 diabetes (T2D), metabolic dysfunction-associated steatotic liver disease (MASLD) and aging. FGF21 and GDF15 also share overlapping regulatory pathways and complementary functions, suggesting a potential coordinated regulation and functional interplay. However, the extent and physiological relevance of this possible interrelationship remain incompletely understood. This review summarizes current knowledge on the regulation, tissue sources, and signaling mechanisms of FGF21 and GDF15 across metabolic diseases and aging, with particular attention to their roles as mediators of adaptive metabolic responses and their overlapping biological actions. We further discuss their context-dependent effects under acute and chronic stress and their relevance as biomarkers of metabolic dysfunction and disease progression. Collectively, this work highlights FGF21 and GDF15 as integrated stress signals linking cellular stress to systemic metabolic adaptation, providing a conceptual framework for understanding their cooperative roles and regulation in metabolic and age-related diseases, which may have therapeutic potential.

## Introduction

Metabolic diseases and age-related disorders represent an increasing global health challenge in the 21^st^ century, accounting for a substantial proportion of morbidity, mortality, and healthcare expenditure worldwide [1, 2]. Age-related disorders encompass a broad spectrum of chronic conditions, including cardiovascular and neurodegenerative diseases, cancer, osteoporosis, and sarcopenia [3, 4]. Metabolic diseases, including obesity, type 2 diabetes (T2D) and metabolic dysfunction-associated steatotic liver disease (MASLD), are also closely associated with aging [5]. Over the last three decades, the burden of these common metabolic diseases has risen markedly [1, 2], emphasizing the urgent need to understand the molecular mechanisms underlying metabolic dysfunction and aging in order to identify novel biomarkers and therapeutic strategies to preserve metabolic health.

A common feature linking aging and metabolic diseases is the progressive accumulation of cellular stress [3, 6]. Several hallmarks of aging, including chronic inflammation, mitochondrial dysfunction, and cellular senescence, are key drivers of metabolic disease [3, 7]. These stress responses activate adaptive signaling pathways aimed at restoring metabolic homeostasis. However, persistent or unresolved stress promotes chronic low-grade inflammation, ectopic fat deposition, and insulin resistance, contributing to progressive metabolic dysfunction, which can also accelerate biological aging [8–10].

**Fig. 1:**
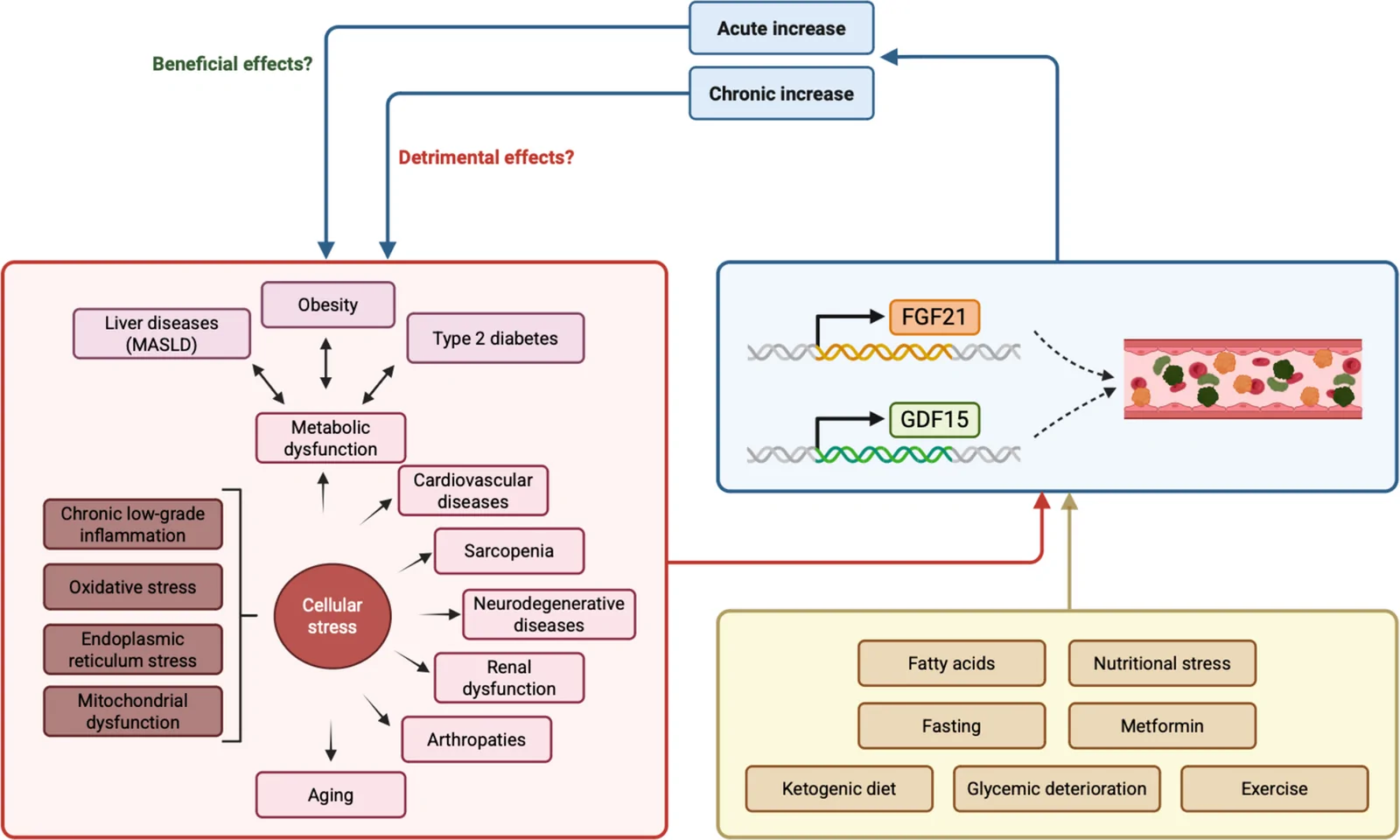
Overview of key pathways and factors that activate cellular stress responses, resulting in elevated circulating FGF21 and GDF15 concentrations. Cellular stress is a central driver of the pathophysiology of metabolic and age-related disorders, with metabolic disturbances further amplifying conditions such as liver diseases, obesity and type 2 diabetes (T2D). These stresses, together with physiological and pharmacological stimuli, increase circulating FGF21 and GDF15 concentrations, potentially contributing to the restoration of metabolic homeostasis. However, the biological significance of their increase may be context-dependent, as acute elevations likely reflect adaptive responses, whereas chronic elevations may indicate sustained metabolic stress and impaired homeostasis. FGF21, fibroblast growth factor 21; GDF15, growth differentiation factor 15; MASLD, metabolic dysfunction-associated steatotic liver disease. Created using BioRender

Among the endocrine mediators activated by cellular stress, fibroblast growth factor 21 (FGF21) and growth differentiation factor 15 (GDF15) have emerged as key stress-responsive cytokines [11]. Although initially investigated independently, accumulating evidence suggests that FGF21 and GDF15 respond to common metabolic and cellular stressors and share overlapping biological functions, raising the possibility that they may act in a coordinated manner to orchestrate adaptive responses to restore metabolic homeostasis. However, it remains unclear whether their induction during metabolic disease reflects adaptive or compensatory responses, and whether their effects are beneficial or detrimental depending on the context (Fig. 1). Additionally, despite their similar regulation under comparable stress conditions, it remains unclear whether these overlapping roles reflect a functional interrelationship, as well as whether combined modulation of both proteins could elicit synergistic therapeutic effects in metabolic diseases. Therefore, this review synthesizes current evidence supporting the coordinated regulation of FGF21 and GDF15, their overlapping biological functions, and their potential functional interplay during metabolic stress and aging, considering their interplay as encompassing the potential convergence and coordination of FGF21 and GDF15 responses to shared stressors, their potential complementary effects on systemic homeostasis, and the possibility that their actions may influence each other directly or indirectly.

## Biological features and regulation of FGF21 and GDF15

**Fig. 2:**
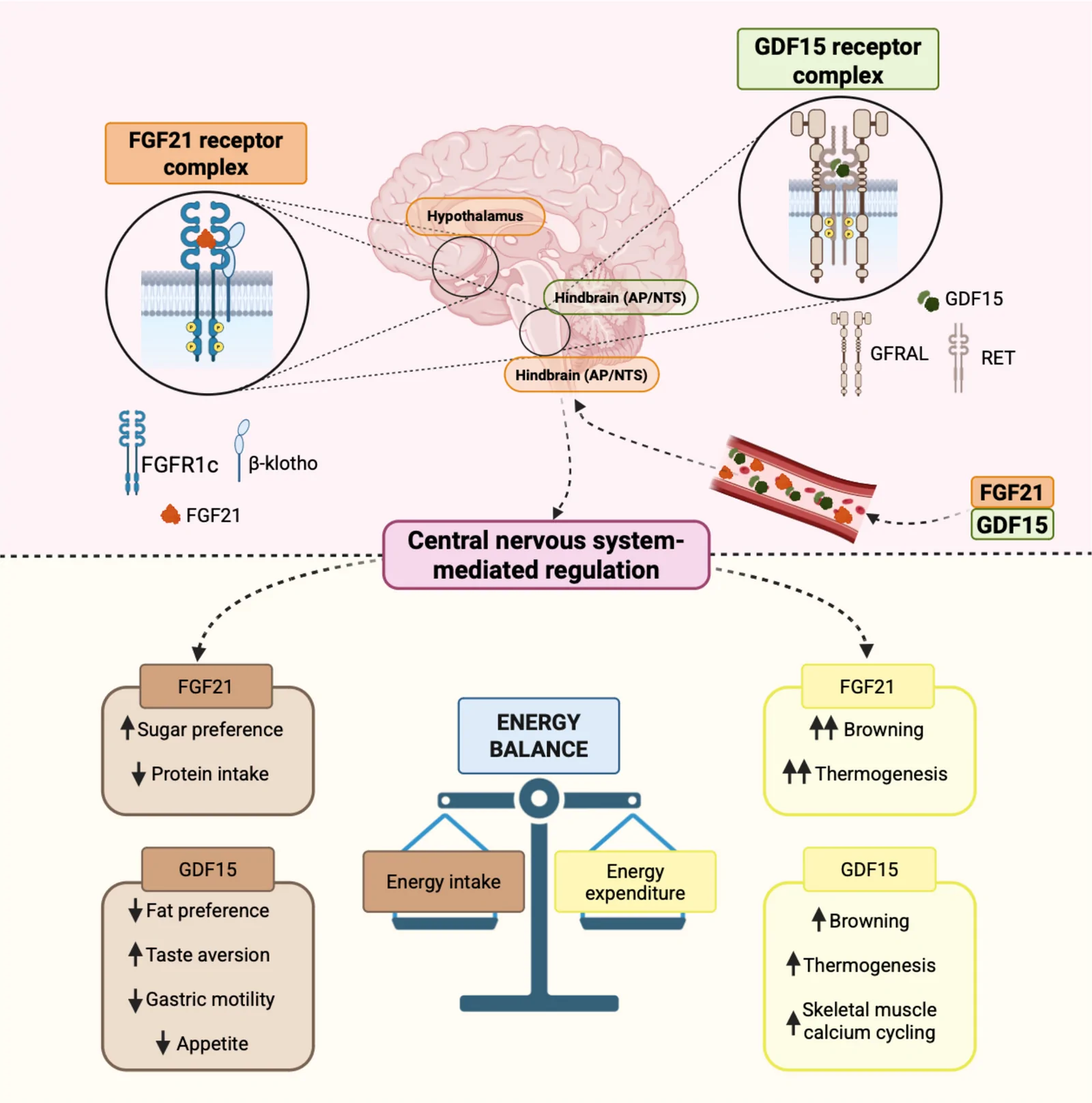
Main roles of FGF21 and GDF15 in the regulation of energy balance. Circulating FGF21 and GDF15 signal to distinct brain regions involved in the control of energy homeostasis. FGF21 is a monomeric protein that primarily acts through FGFR1c/β-klotho-expressing neurons within hypothalamic regions, but also through neuronal populations in the area postrema (AP) and nucleus of the solitary tract (NTS) of the hindbrain; whereas GDF15 is a dimeric protein that signals through the GFRAL/RET receptor complex, which is selectively expressed in the AP and NTS of the hindbrain. Activation of these pathways modulates feeding behavior, nutrient preference, thermogenesis, adipose tissue browning and energy expenditure, thereby contributing to the regulation of whole-body energy balance. FGF21, fibroblast growth factor 21; GDF15, growth differentiation factor 15; FGFR1c, fibroblast growth factor receptor 1c; GFRAL, glial-derived neurotrophic factor receptor alpha-like; RET, rearranged during transfection; AP, area postrema; NTS, nucleus of the solitary tract. Created using BioRender

FGF21 was identified in 2000 as a novel member of the FGF family [12]. To exert its effect, FGF21 binds to fibroblast growth factor receptors (FGFRs), mainly to the FGFR1c isoform, which requires the presence of the β-klotho co-receptor for signaling. FGF21 is expressed in various tissues, including liver, adipose tissue, skeletal muscle, pancreas, cardiac muscle, and brain [13]. Although its receptors display a broad tissue distribution, FGFR1c exhibits a ubiquitous expression pattern, whereas β-klotho (encoded by the *KLB* gene) is predominantly expressed in metabolic tissues, including adipose tissue, liver, and pancreas, with these organs being the primary sites of FGF21 action. Additionally, β-klotho is also expressed in specific neuronal populations within the hypothalamus and in the area postrema (AP) and nucleus of the solitary tract (NTS) of the hindbrain, albeit at lower levels than in metabolic tissues, contributing to the systemic effects of FGF21 [13–15] (Fig. 2). Circulating FGF21 concentrations are normally very low under healthy conditions [16] but have been shown to increase in response to several stressors, including mitochondrial stress, nutritional challenges such as obesity, fasting, malnutrition, or T2D [17] as well as age-related diseases (Fig. 1), highlighting its potential as a target for therapeutic strategies [18].

GDF15 was discovered in 1997 by three independent research groups [19–21] as a member of the transforming growth factor β (TGF-β) superfamily [22] and exerts its effects through the interaction with the glial cell-derived neurotrophic factor family receptor α-like (GFRAL) protein and the tyrosine kinase co-receptor rearranged during transfection (RET) [23–26] (Fig. 2). Although *GFRAL* expression was initially considered largely restricted to the brainstem [23–26], a number of studies have reported low-level detection in peripheral tissues such as the testes and white adipose tissue (WAT) [25, 27]. These findings have sparked ongoing discussion about whether GFRAL is expressed in peripheral tissues or whether unknown GDF15 receptors exist and mediate its peripheral effects [27]. Based on current knowledge, it is plausible that additional receptors for GDF15, currently unidentified, mediate its actions in peripheral tissues. Meanwhile, GDF15 is expressed in multiple tissues under physiological conditions, including the liver, intestine, kidneys, and placenta. Despite this widespread expression, its circulating levels are normally very low and increase during stress conditions [22]. Consequently, GDF15 has received considerable attention due to its association with numerous biological processes and diseases, many of which are age-related disorders [28]. These conditions include inflammation, insulin resistance, fibrogenesis, mitochondrial dysfunction, cancer, chronic kidney diseases, cardiovascular diseases, and obesity, among others [28, 29] (Fig. 1).

Overall, FGF21 and GDF15 share several features as stress-responsive endocrine mediators. Both are induced by metabolic and cellular stress and circulate at low concentrations under basal physiological conditions, with their levels increasing across a broad range of metabolic and age-related conditions. Their induction involves partially overlapping upstream pathways, including the integrated stress response (ISR), as well as other transcriptional programs activated by mitochondrial, endoplasmic reticulum (ER), and metabolic stress. However, they differ in their receptor systems, tissue source and targets, main signaling pathways and specific downstream effects, which may contribute to their overlapping yet distinct roles in metabolic adaptation and disease (Table 1, Fig. 2).

**Table 1:** Comparative overview of FGF21 and GDF15

Feature	FGF21	GDF15
Main receptor	FGFR1c/β-klotho. Broadly expressed, mainly in metabolic tissues	GFRAL/RET. Apparently restricted to the brainstem
Main tissue source	Liver/adipose tissue (depending on the context)	Liver/adipose tissue (depending on the context)
Main target tissues	Liver, adipose tissue, pancreas, CNS	Primarily GFRAL-expressing neurons in the area postrema/NTS
Main inducers	Changes in nutrient availability, cold exposure, cellular stress, inflammation	Cellular stress, inflammation, aging
Major signalling pathways	PPARα, AMPK, ATF4	ISR/UPR activation, ATF4/CHOP

*FGF21* fibroblast growth factor 21, *GDF15* growth differentiation factor 15, *FGFR1c* fibroblast growth factor receptor 1c, *GFRAL* glial cell-derived neurotrophic factor family receptor α-like, *RET* tyrosine kinase co-receptor rearranged during transfection, *CNS* central nervous system, *NTS* nucleus of the solitary tract; *ISR*, integrated stress response, *UPR* unfolded protein response, *PPARα* peroxisome proliferator-activated receptor α, *AMPK* adenosine monophosphate-activated protein kinase, *ATF4* activating transcription factor 4, *CHOP* C/EBP homologous protein

## Core metabolic biological functions of FGF21 and GDF15

The regulation of energy balance involves a complex interplay between energy intake and energy expenditure, ensuring metabolic stability [30]. Over the past decades, both FGF21 and GDF15 have been extensively investigated because of their critical roles in maintaining energy homeostasis [13, 31]. Both cytokines have emerged as promising therapeutic candidates for the treatment of metabolic complications, given their involvement in the regulation of glucose, lipid, and energy metabolism, thereby influencing insulin sensitivity and glucose tolerance. However, although both proteins can modulate energy balance, FGF21 primarily promotes energy expenditure by enhancing thermogenesis and changing food preferences [13, 32], whereas GDF15 mainly acts by suppressing food intake [31] (Fig. 2). Therefore, their effects may contribute to the coordinated regulation of energy balance in response to different metabolic conditions through complementary actions.

### Glucose homeostasis

Both FGF21 and GDF15 have been implicated in the regulation of glucose homeostasis and insulin sensitivity, although they appear to act through distinct signaling pathways. FGF21 administration lowers circulating glucose and insulin concentrations, thereby improving glucose tolerance and insulin sensitivity [33, 34]. A single injection of FGF21 has been shown to markedly decrease plasma glucose levels in *ob/ob* and diet-induced obese (DIO) mice, supporting its role as an insulin sensitizer [33]. Exposure of adipocytes to recombinant FGF21 activates mitogen-activated protein kinase (MAPK) and downstream extracellular signal-regulated protein kinase 1 and 2 (ERK1/2), which increase the expression of glucose transporter-1 (GLUT1), thereby stimulating glucose uptake [35, 36]. Consistent with a central role of adipose tissue in the insulin-sensitizing effects of FGF21, adipose-specific *Klb*-knockout (KO) mice show an impaired response to the coadministration of FGF21 and insulin compared with wild type (WT) controls [37]. Thus, FGF21 appears to enhance insulin sensitivity, at least in part, through direct signaling in WAT. Whether adiponectin mediates the effects of FGF21 on glucose homeostasis and insulin sensitivity remains under debate. Some studies have demonstrated that FGF21 administration induces adiponectin expression and secretion in adipocytes, increasing circulating adiponectin concentrations, and that this response is required for its glucose-lowering and insulin-sensitizing effects [14, 38, 39]. In contrast, other studies have reported that although FGF21 signaling to WAT is required for acute insulin sensitization, FGF21 administration does not increase plasma adiponectin levels, indicating that adiponectin may not be required for the metabolic effects of FGF21 [37]. These discrepancies may reflect differences in experimental conditions, including differences in high-fat diet (HFD) exposure duration, FGF21 dose and timing, or age.

GDF15 also improves insulin sensitivity, although via a distinct pathway. Mice lacking *Gdf15* and/or *Gfral* are more susceptible to insulin resistance [25, 40]. GDF15 has been shown to enhance insulin action in the liver, brown adipose tissue (BAT) and WAT through GFRAL-dependent β_1,2_-adrenergic signaling [41], independently of changes in food intake and weight loss [41, 42]. Thus, although FGF21 and GDF15 converge on the regulation of insulin sensitivity, FGF21 acts predominantly through WAT signaling, whereas GDF15 acts through central signaling to modulate glucose metabolism in peripheral tissues. Importantly, mice with combined whole-body deletion of *Fgf21* and *Gdf15* exhibited greater insulin resistance than *Gdf15* single KO mice [43], suggesting a synergistic role for both cytokines in maintaining glucose homeostasis. Paradoxically, despite their beneficial metabolic effects, circulating concentrations of FGF21 and GDF15 are increased in insulin-resistant conditions, including obesity, T2D and liver diseases, which may reflect a compensatory response to metabolic stress or impaired responsiveness to FGF21 and GDF15 signaling rather than improved metabolic function [11].

### Food preference and appetite regulation

FGF21 and GDF15 regulate the energy balance by modulating energy intake. However, they appear to influence feeding behavior through distinct but potentially complementary mechanisms. FGF21 is particularly involved in the regulation of macronutrient preference, whereas GDF15 primarily acts as an anorectic signal that suppresses food intake [29, 44] (Fig. 2). Human genetic studies have identified single-nucleotide polymorphisms within the *FGF21* sequence that are associated with carbohydrate consumption [45, 46], particularly with increased intake of sweets and alcohol [47]. Both acute and chronic carbohydrate intake induce FGF21 expression [48, 49], and elevated circulating FGF21 concentrations have been observed in humans [47] and mice [50, 51] displaying a reduced preference for sugar. This increase in FGF21 levels is associated with an increase in hepatic *Fgf21* mRNA expression driven by the carbohydrate-responsive element-binding protein (ChREBP) transcription factor [52, 53]. FGF21 exerts these effects by signaling to glutamatergic neurons in the hypothalamus, which suppresses carbohydrate preference without affecting lipid or protein intake [52, 54]. Protein restriction also induces hepatic *FGF21* expression, increasing circulating FGF21 concentrations that signal to the brain to modulate macronutrient preference [55–58]. Higher FGF21 levels in humans are associated with lower protein intake, suggesting that low protein availability promotes FGF21 secretion [59]. In this sense, it was initially proposed that elevated FGF21 levels under protein restriction may promote a shift toward greater protein intake [60, 61]. However, findings by Flippo et al. suggested that rather than specifically enhancing protein preference, FGF21 suppresses sugar intake, thereby promoting the search for alternative macronutrients [62]. Notably, the rise in FGF21 concentrations induced by protein restriction is required for several metabolic benefits, including increased energy expenditure, reduced weight gain and improved glucose homeostasis in DIO mice, as these effects are blunted in mice lacking *Fgf21* [60]. Interestingly, although protein intake appears to be the primary determinant of circulating FGF21 levels, the maximal FGF21 elevation is observed in mice fed low-protein and high-carbohydrate diets [63]. Consistent with this, co-ingestion of protein with sucrose suppresses the postprandial FGF21 secretion typically induced by carbohydrate consumption, further supporting proteins as a key modulator of FGF21 regulation [64].

In contrast to the specific effects of FGF21 on macronutrient preference, GDF15 primarily reduces body weight through appetite suppression [29]. GDF15 can additionally alter food preferences, particularly reducing the preference for HFD [65, 66] (Fig. 2). This effect requires its receptor, GFRAL, as it is absent in *Gfral*-deficient mice [65]. Activation of the ISR in BAT and WAT induces GDF15 secretion, which in turn suppresses HFD consumption [66]. Further studies have shown that fatty acids increase GDF15 levels and reduce food intake in WT mice through the GDF15-GFRAL axis, as this effect disappears in mice lacking *Gfral* [67, 68]. GDF15 also induces aversive and gastrointestinal responses that further distinguish its effects from those of FGF21 [69]. GDF15 causes emesis in musk shrews and induces nausea-like behaviors in mice and rats [70]. Moreover, recombinant GDF15 delays gastric emptying in mice, probably through a vagal-mediated mechanism [65]. At the central level, GDF15 activates ERK1/2 and signal transducer and activator of transcription 3 (STAT3) signaling in the hypothalamus, suppressing neuropeptide Y (NPY) and activating pro-opiomelanocortin (POMC), thereby reducing food intake and body weight [71]. Consistent with these findings, lack of *Gfral* in the AP/NTS increased WAT mass and body weight in mice fed a HFD [72]. Likewise, mice lacking *Gdf15* or *Gfral* exhibit increased food intake, adiposity, insulin resistance, and glucose intolerance when fed a HFD, whereas these effects are not observed when they are fed a normal chow diet compared with WT mice [23–26]. Some authors have proposed that the actions of GDF15 may be enhanced during obesity through its interaction with other activated pathways, suggesting that the physiological effects of GDF15 are strongly dependent on metabolic context. Co-administration of GDF15 and leptin during obesity appears to potentiate the effects of GDF15 in reducing food intake and body weight. However, leptin administration alone did not reduce body weight in obese mice, despite their usually elevated endogenous GDF15 levels [73]. This suggests that the increase in endogenous GDF15 is insufficient to potentiate leptin’s effects, while its pharmacological administration or overexpression may produce a stronger activation of GFRAL-RET-mediated pathways, leading to metabolic improvements.

Overall, the distinct effects of FGF21 and GDF15 on feeding behavior may allow them to regulate energy intake in a coordinated manner. FGF21 primarily modifies the composition of food intake by reducing preference for carbohydrates, whereas GDF15 predominantly suppresses overall food intake through several mechanisms, including reduced HFD preference and aversive and gastrointestinal responses. Thus, their actions may be functionally complementary, allowing coordinated regulation of the composition and amount of food intake in response to different metabolic challenges.

### Energy expenditure and thermogenesis

Thermogenesis, the process of heat production primarily through metabolic processes, contributes to energy expenditure and body temperature regulation [74]. Adaptive thermogenesis is stimulated by cold exposure or dietary intake and is primarily mediated by BAT and WAT containing beige adipocytes [75, 76]. Both brown and beige adipocytes express uncoupling protein 1 (UCP1), a mitochondrial protein that facilitates the dissipation of energy as heat by uncoupling oxidative phosphorylation [75]. Cold exposure activates the sympathetic nervous system (SNS), leading to the stimulation of β-adrenergic receptors in brown adipocytes. This process induces *UCP1* expression, promoting thermogenesis and browning of WAT, ultimately increasing energy expenditure to help maintain energy homeostasis [77]. Within this adaptive response, FGF21 and GDF15 have emerged as metabolically active mediators that may contribute to the regulation of energy expenditure through both overlapping and distinct mechanisms.

Both FGF21 and GDF15 are induced in BAT and WAT during cold exposure and have been associated with increased thermogenic activity [78–83]. SNS activation by cold exposure triggers the release of norepinephrine, which, via β-adrenergic receptors, stimulates *Fgf21* expression in BAT through cyclic adenosine monophosphate (cAMP)-mediated pathways, thereby increasing plasma FGF21 concentrations [80]. Cold exposure also triggers the ISR response, increasing protein levels of activating transcription factor 4 (ATF4), which induces *Fgf21* and *Gdf15* expression [82]. Interestingly, three days of cold exposure in mice lacking *Fgf21* in BAT did not affect circulating FGF21 levels, suggesting that BAT-derived FGF21 does not significantly contribute to circulating FGF21 concentrations during short-term cold exposure [82]. The FGF21 response may also depend on the timing of cold exposure, as circulating FGF21 concentrations increase in humans in the evening but not in the morning after cold exposure [84]. Elevated FGF21 levels are also associated with the browning of WAT, as pharmacological administration of FGF21 induces the expression of peroxisome proliferator-activated receptor γ co-activator (PGC) -1α protein and UCP1, consequently enhancing thermogenesis in BAT and promoting the browning of WAT depots (Fig. 2) [79]. Notably, FGF21 still increases energy expenditure in *Ucp1*-KO mice, suggesting that FGF21 promotes thermogenesis through both UCP1-dependent and -independent mechanisms [85]. Reduced energy intake has also been observed in *Ucp1*-KO mice following FGF21 treatment, which may represent an alternative mechanism contributing to its thermogenic effects independently of UCP1 [86, 87].

In contrast to FGF21, GDF15 may extend this adaptive response beyond adipose tissue. Increased *Gdf15* expression has been associated with enhanced expression of thermogenic and lipolytic genes in BAT and WAT, conferring resistance to obesity [88]. Consistent with these findings, GDF15 treatment enhances energy expenditure, with an upregulation of *Ucp1* expression in inguinal WAT (iWAT) of DIO mice, leading to reduced body weight and adiposity [89]. Thus, like FGF21, GDF15 can promote thermogenic activation in adipose tissue, contributing to increased energy expenditure. However, GDF15 treatment also promotes energy expenditure in the skeletal muscle of mice through a GFRAL/β-adrenergic signaling axis, sustaining fatty acid oxidation and calcium futile cycling, which helps to prevent the reductions in energy expenditure during caloric restriction [90]. Therefore, although both mediators can promote energy expenditure and thermogenic activation, FGF21 appears to be more closely associated with BAT activation and WAT browning, whereas GDF15 may additionally engage skeletal muscle to regulate energy expenditure through mechanisms that are not restricted to UCP1-dependent thermogenesis.

Importantly, evidence suggests that FGF21 and GDF15 may also interact during the response to sympathetic stimulation. Campderrós et al. [83] showed that FGF21 is required for GDF15 induction in brown and beige adipocytes following β_3_-adrenergic stimulation, as the absence of *Fgf21* or *Klb* impairs *Gdf15* expression (Table 2). These findings suggest that FGF21 may act upstream of GDF15 within adipose tissue during thermogenic activation, providing a potential mechanistic link between them. However, this relationship appears to be tissue- and context-dependent. Hepatic-specific *Fgf21* deletion did not prevent β_3_-adrenergic induction of *Gdf15* expression (Table 2) [91], while BAT-specific *Fgf21* deletion did not alter circulating GDF15 levels after three days of cold exposure (Table 2) [82]. These findings suggest that GDF15 induction can be maintained in the absence of FGF21 from specific tissues, raising the possibility of compensation by FGF21 from other tissues or alternative regulatory pathways. Thus, although FGF21 may act upstream of GDF15 under some conditions, their relationship does not appear to be strictly linear. Moreover, Mendez-Gutierrez et al. [92] reported an increase in circulating levels of both FGF21 and GDF15 in humans after two hours of cold exposure; however, only FGF21 levels were associated with BAT volume, suggesting a closer relationship between FGF21 and BAT activation under these conditions.

**Table 2:** Experimental evidence for context-dependent interactions between FGF21 and GDF15

Intervention	System/Conditions	Effect on FGF21	Effect on GDF15	Possible interpretation	Reference
*Gdf15*-KO	20 weeks HFD Male mice	↑ Hepatic mRNA expression ↑ Circulating levels	-	Compensatory or stress-induced response	Patel et al. (2022) [43]
*Fgf21*-KO	20 weeks HFD Male mice	-	↑ Circulating levels		
Hepatic-specific *Gdf15* deletion	12 weeks HFD Male mice	↑Circulating levels	-		
β_3_-adrenergic stimulation	Brown and beige mice adipocytes	↑ Expression	↑ Expression	*Fgf21* is required for *Gdf15* induction in response to β_3_-adrenergic stimulation	Campderros et al. (2019) [83]
β_3_-adrenergic stimulation	Brown and beige mice adipocytes *Fgf21/Klb* KO	-	No changes		
β_3_-adrenergic stimulation	Hepatic *Fgf21* deletion DIO mice	-	↑Circulating levels	Hepatic *Fgf21* is not essential for *Gdf15* induction in response to β_3_-adrenergic stimulation	Mori et al. (2026) [91]
3 days of cold exposure in mice	Mice with BAT-specific *Fgf21* deletion	-	No changes	BAT *Fgf21* is not essential for *Gdf15* induction in response to cold	Bjorkman et al. (2024) [82]
Hepatic *Gdf15* overexpression	HFD mice	↑ Hepatic mRNA expression ↑ Circulating levels	↑ Hepatic mRNA expression ↑ Circulating levels	Unidirectional regulatory relationship	Takeuchi et al. (2024) [93]
Hepatic *Fgf21* overexpression	HFD mice	↑ Hepatic mRNA expression ↑ Circulating levels	No changes		

*FGF21* fibroblast growth factor 21, *GDF15* growth differentiation factor 15, *KO* knockout, *Klb* β-klotho, *DIO* diet-induced obesity, *HFD* high-fat diet, *BAT* brown adipose tissue; ↑, indicates an increase; -, indicates no effect

Overall, FGF21 and GDF15 appear to participate in a shared adaptive response to cold-induced metabolic stress, but their contributions to thermogenesis are not fully redundant. FGF21 is more closely linked to BAT activation and WAT browning, whereas GDF15 may complement these effects by promoting energy expenditure through skeletal muscle (Fig. 2). Moreover, the ability of β-adrenergic stimulation to induce GDF15 through FGF21-dependent and FGF21-independent mechanisms suggests that their interaction is tissue- and context-dependent and may involve compensatory pathways. Thus, FGF21 and GDF15 may converge on the maintenance of energy expenditure while engaging partially distinct but potentially complementary tissue-specific mechanisms.

## FGF21 and GDF15 in obesity and metabolic diseases

Obesity represents a major metabolic disorder and a major risk factor for the development of metabolic diseases [94, 95]. The chronic metabolic disturbances associated with obesity can progressively impair glucose and lipid homeostasis and contribute to the development of obesity-related complications [95]. Consistent with this, the increasing prevalence of obesity has been accompanied by a rising prevalence of several metabolic disorders, including T2D, one of the most strongly obesity-associated complications [96, 97], in which obesity-induced insulin resistance represents a key feature preceding its onset [98], as well as nonalcoholic fatty liver disease (NAFLD), now termed MASLD, and its progressive form, metabolic dysfunction-associated steatohepatitis (MASH) [99].

Obesity, T2D, and MASLD/MASH are associated with profound alterations in metabolic homeostasis, cellular stress, and inflammatory responses that can modulate the expression of FGF21 and GDF15 [11, 100]. Although both factors are involved in adaptive responses to metabolic stress and the regulation of glucose and lipid homeostasis, their circulating levels and biological effects may vary depending on the underlying disease context. This has prompted increasing interest in their roles as potential biomarkers and therapeutic targets in these conditions.

### FGF21 and GDF15 in obesity

**Fig. 3:**
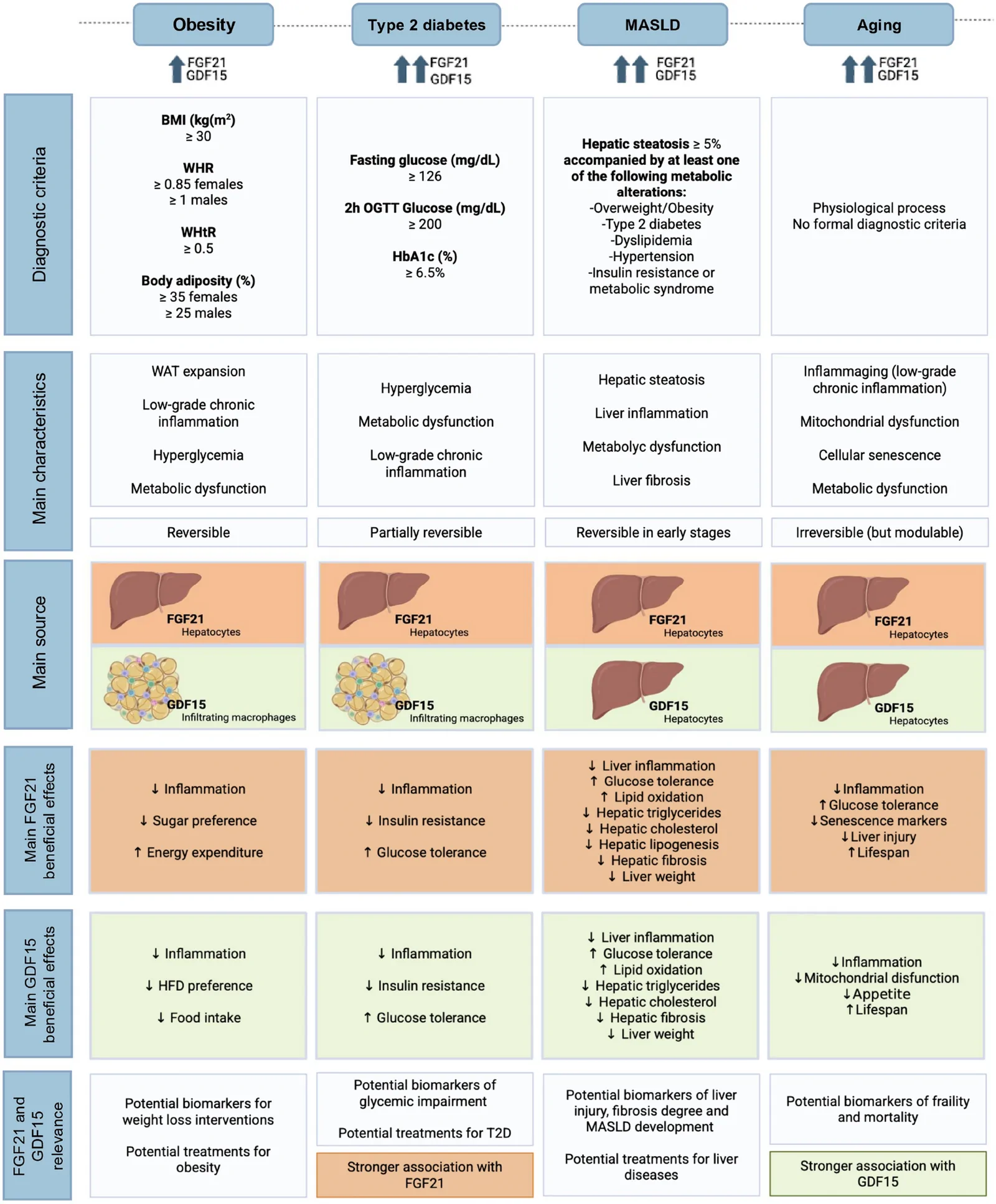
Comparative role of FGF21 and GDF15 across obesity, type 2 diabetes (T2D), metabolic dysfunction-associated steatotic liver disease (MASLD) and aging. The figure summarizes the diagnostic criteria and the main characteristics of obesity, T2D, MASLD and aging. Additionally, it resumes the major tissue sources, beneficial effects and potential clinical relevance of FGF21 and GDF15 across these conditions. Although circulating levels of FGF21 and GDF15 are consistently increased in obesity, T2D, MASLD and aging, their predominant tissue sources may differ depending on the underlying pathological context. Furthermore, both stress-induced cytokines exert pleiotropic effects that contribute to the maintenance of metabolic homeostasis, highlighting their emerging value as biomarkers of disease progression and as promising therapeutic targets for metabolic and age-related disorders. BMI, body mass index; FGF21, fibroblast growth factor 21; GDF15, growth differentiation factor 15; WHR, waist-to-hip ratio; WHtR, waist-to-height ratio; OGTT, oral glucose tolerance test; HbA1c, hemoglobin A1c; WAT, white adipose tissue; HFD, high-fat diet. Created using BioRender

Obesity is characterized by chronic nutrient excess and progressive disruption of metabolic homeostasis, which promote several forms of cellular stress, including oxidative stress, ER stress, mitochondrial dysfunction, and chronic low-grade inflammation [101, 102]. These stressors activate adaptive endocrine responses aimed at restoring metabolic and tissue homeostasis, among which FGF21 and GDF15 have emerged as important stress-responsive mediators. In fact, GDF15 has been highlighted as one of the best markers of systemic stress [103]. Cellular stress activates the ISR, including the protein kinase R-like ER kinase (PERK)/eukaryotic initiation factor 2 α (eIF2α)/ATF4 and C/EBP homologous protein (CHOP) pathways [104], which increase the expression of FGF21 [105–107] and GDF15 [108–110]. Through these stress-responsive pathways, local cellular disturbances can be translated into endocrine signals that coordinate systemic metabolic adaptation. In this context, FGF21 and GDF15 may act as endocrine mediators linking tissue-specific stress responses with whole-body regulation of energy balance, inflammation and metabolic homeostasis. Consistently, circulating concentrations of FGF21 and GDF15 are elevated in both mice and humans with obesity (Figs. 1, 3) [65, 111, 112]. Importantly, their elevation may reflect a compensatory response to the metabolic and cellular stress associated with sustained caloric excess, rather than necessarily indicating a pathogenic role. Consistent with a protective role for endogenous GDF15, *Gdf15*-KO mice are more prone to DIO than WT mice, developing worse glucose tolerance [40]. Likewise, although FGF21 concentrations are increased during obesity, pharmacological administration of FGF21 improves metabolic parameters and promotes weight loss [113], suggesting that the endogenous increase may be insufficient to fully counteract the metabolic disturbances associated with chronic overnutrition. Notably, a chronic state of caloric excess is necessary to increase circulating GDF15 levels rather than short-term overfeeding [108].

**Fig. 4:**
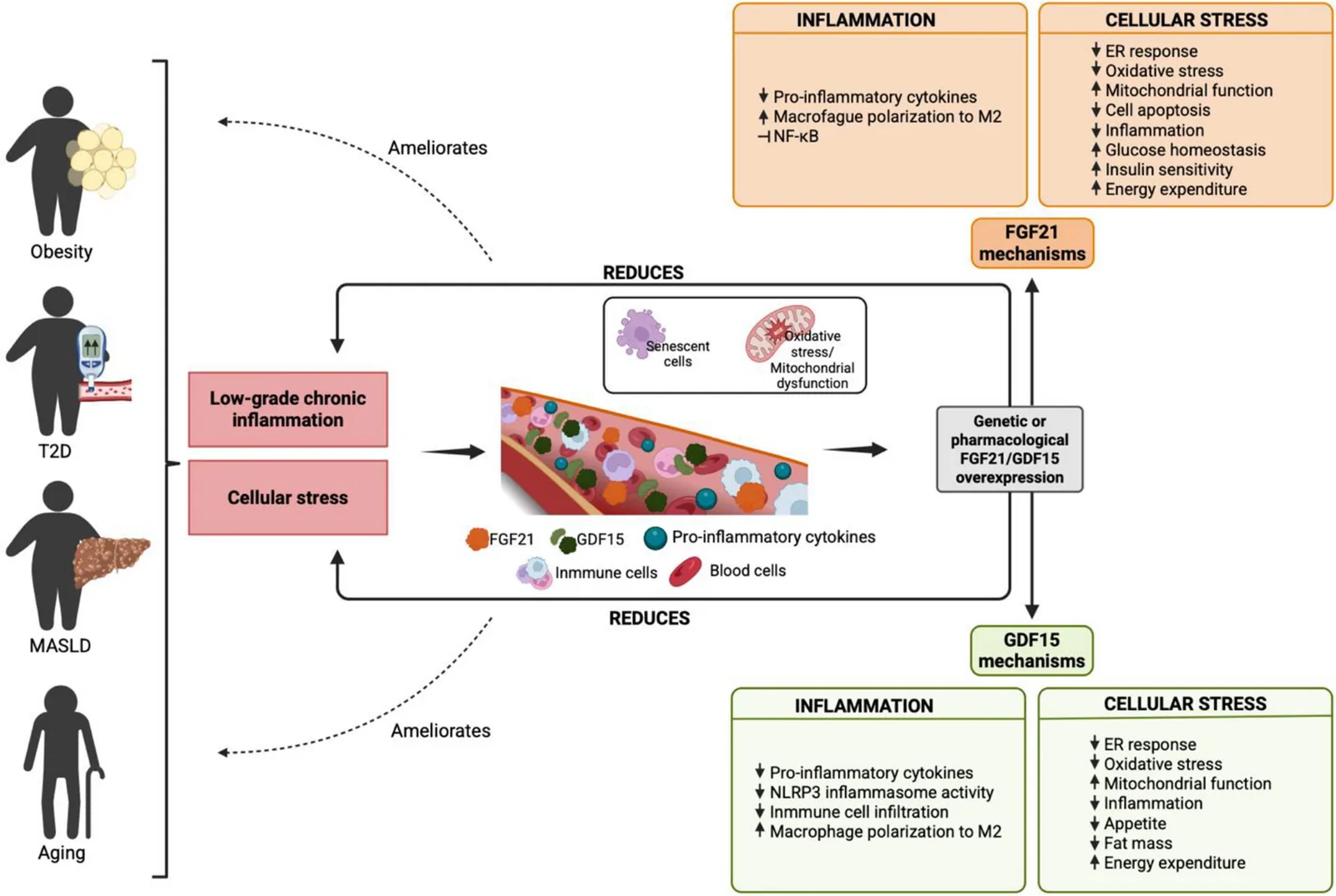
Main effects of FGF21 and GDF15 on inflammation and cellular stress. Obesity, type 2 diabetes (T2D), metabolic dysfunction-associated steatotic liver disease (MASLD) and aging, are characterized by chronic low-grade inflammation and cellular stress, which increase circulating FGF21 and GDF15 concentrations. Experimental genetic or pharmacological overexpression of FGF21 and GDF15 attenuates inflammation and cellular stress through complementary mechanisms, contributing to improved age-related diseases and healthier aging. FGF21, fibroblast growth factor 21; GDF15, growth differentiation factor 15; NF-κB, nuclear factor kappa B, ER, endoplasmic reticulum; NLRP3, NOD-like receptor pyrin domain-containing protein 3. Created using BioRender

Interestingly, FGF21 has been shown to reduce ER and oxidative stress, improve mitochondrial function and promote cell survival (Fig. 4) through signaling pathways including AMP-activated protein kinase/sirtuin 1/peroxisome proliferator-activated receptor gamma coactivator 1-α (AMPK/SIRT1/PGC-1α), phosphoinositide 3-kinase/protein kinase B (PI3K/AKT) and nuclear factor erythroid 2-related factor (NRF2) [106, 114–118]. Likewise, GDF15 contributes to systemic metabolic adaptations by improving fatty acid oxidation and reducing ER and oxidative stress through an AMPK-dependent mechanism, as well as reducing food intake, inducing WAT browning and improving insulin sensitivity (Fig. 4) [119–121]. In parallel, both factors appear to exert anti-inflammatory effects that contribute to the maintenance of tissue and systemic homeostasis (Fig. 4). FGF21 has been shown to reduce pro-inflammatory cytokine expression, inhibit NF-κB signaling [122–125] and promote macrophage polarization toward an anti-inflammatory M2 phenotype (Fig. 4) [126–128]. However, inflammatory conditions can reduce *Klb* expression, potentially limiting FGF21 responsiveness despite elevated circulating levels [129–132], with NOD-like receptor pyrin domain-containing protein 3 (NLRP3) inflammasome activity, a central driver of obesity-induced inflammation and insulin resistance, potentially contributing to this dysregulation [130, 133, 134]. Similarly, GDF15 reduces systemic and tissue inflammation, partly by decreasing pro-inflammatory cytokines, macrophage infiltration and NLRP3 inflammasome activity [135–138], and by promoting anti-inflammatory macrophage polarization (Fig. 4) [139, 140]. In addition, it has been proposed that GDF15 mediates improvements in metabolic health and may indirectly reduce inflammation through decreases in adiposity rather than direct action on immune cells [89, 136, 137]. Together, these findings suggest that FGF21 and GDF15 act as components of an integrated adaptive response to obesity-associated cellular stress, linking stress sensing to endocrine, metabolic and inflammatory pathways to maintain systemic homeostasis.

Additionally, determining the tissue-specific contribution to circulating FGF21 and GDF15 levels during obesity is challenging, because long-term HFD feeding induces multiple metabolic disturbances across multiple organs. In mice, HFD increases *Gdf15* expression predominantly in epididymal WAT (eWAT) and, to a lesser extent, in the liver. Moreover, circulating GDF15 concentrations correlate positively with *Gdf15* expression in eWAT, but not in the liver, suggesting that eWAT is a major source of circulating GDF15 levels during obesity. Consistent with this, individuals with obesity exhibit increased *GDF15* expression in visceral adipose tissue (VAT), associated with macrophage infiltration, whereas hepatic expression remains unchanged [141]. In contrast, the liver is considered the main source of circulating FGF21 concentrations during obesity [142, 143], although the contribution of WAT remains controversial. While subcutaneous adipose tissue (scWAT) does not appear to contribute to circulating FGF21 levels in humans [144], some studies have reported a positive association between subcutaneous fat and circulating FGF21 concentrations, along with increased *Fgf21* mRNA expression in the liver, BAT and WAT of mice [112]. At the cellular level, FGF21 is predominantly expressed in adipocytes within WAT, whereas GDF15 expression is primarily derived from infiltrating macrophages in WAT [108, 141] and hepatocytes in the liver [43, 141]. These findings indicate that WAT is a major source of GDF15 early in obesity and T2D [141], whereas the liver remains the primary contributor to circulating FGF21 [145] (Fig. 4). Moreover, FGF21 and GDF15 expression during obesity also shows sex differences. GDF15 levels are increased in men with obesity compared to women with obesity [146, 147], whereas FGF21 concentrations are also generally higher in men, although the evidence is less consistent [146, 148]. Therefore, these differences may impact metabolic responses and should be considered in research and therapeutic strategies [149, 150].

Overall, obesity-associated metabolic dysfunction can be viewed as a state of chronic cellular stress that activates an integrated FGF21- and GDF15-mediated adaptive response. Both factors can contribute to the restoration of metabolic and tissue homeostasis by counteracting cellular stress, inflammation and metabolic imbalance. However, persistent nutrient excess and unresolved stress may alter these compensatory responses, as suggested by the coexistence of elevated circulating FGF21 and GDF15 concentrations with metabolic dysfunction. Thus, elevated levels of FGF21 and GDF15 may reflect an ongoing attempt to restore metabolic homeostasis that becomes insufficient as metabolic stress persists, with the balance between stress-induced production and tissue responsiveness determining the effectiveness of this adaptive response.

### FGF21 and GDF15 in type 2 diabetes

T2D is driven by insulin resistance and progressive β-cell dysfunction and often remains undiagnosed until complications emerge [151–153]. Given their involvement in glycemic control, FGF21 and GDF15 have attracted attention as metabolic regulators that may influence T2D progression (Fig. 3). Notably, despite the beneficial effects previously discussed of FGF21 on glycemic control, circulating FGF21 levels increase with worsening glycemic status, reaching their highest concentrations in individuals with T2D (Fig. 3) [146, 154], while *Klb* expression is reduced by increased pro-inflammatory cytokines [129–132, 155, 156]. Additionally, FGF21 has been proposed as an early biomarker for the development of T2D. In male rats fed a HFD, circulating FGF21 concentrations increased before insulin sensitivity impairment, suggesting that FGF21 could be an early biomarker of the onset of insulin resistance and metabolic dysfunction [157]. In humans, elevated circulating FGF21 concentrations have been associated with an increasing number of metabolic syndrome components and identified as an independent risk factor for metabolic syndrome [112]. Furthermore, studies in both Caucasian and Chinese populations have shown that circulating FGF21 levels independently predict incident metabolic syndrome, progression to impaired glucose metabolism [158, 159] and future T2D risk before the clinical onset of glucose dysregulation [160].

Similar to FGF21, circulating GDF15 levels are increased in T2D (Fig. 3) and positively correlate with glucose, hemoglobin A1c (HbA1c), homeostatic model assessment of insulin resistance (HOMA-IR), and negatively correlate with quantitative insulin sensitivity check index (QUICKI) [146, 161]. GDF15 has also been proposed as a biomarker of cardiometabolic diseases, including T2D [162]. In people with pre-diabetes or T2D, elevated GDF15 concentrations are associated with impaired glycemic control [161], and increased GDF15 concentrations are detected several years before the manifestation of T2D [163, 164]. A large prospective cohort study showed that individuals who developed T2D had higher baseline GDF15 levels than those who did not. Among those who developed diabetes, higher baseline GDF15 concentrations were associated with shorter diabetes-free survival [165]. Furthermore, GDF15 levels further increase with the progression of diabetes and its complications, suggesting its potential as a biomarker for T2D deterioration [166–168].

Interestingly, a recent study from our group suggests that FGF21 is a better predictor of the presence of T2D than GDF15 (Fig. 3) [146] and proposes the FGF21/adiponectin ratio as a superior biomarker compared with FGF21 alone, in agreement with another study in a Chinese population [146, 169]. Moreover, both FGF21 and GDF15 have been identified as independent predictors of long-term mortality in people with T2D, and they were proposed together with C-X-C motif chemokine ligand 9 (CXCL9) and neurofilament light chain (NFL), as a composite biomarker panel to identify individuals at higher risk of mortality among people with T2D [170].

Overall, FGF21 and GDF15 act as complementary metabolic stress hormones, both elevated in T2D and closely linked to impaired glucose homeostasis. Their elevations precede the onset of T2D, highlighting their potential as early biomarkers of disease development and progression (Fig. 3).

### FGF21 and GDF15 in MASLD/MASH and liver fibrogenesis

MASLD is a major metabolic liver disease characterized by excessive fat accumulation in hepatocytes in the context of metabolic dysfunction and can progress to MASH, which is defined by hepatic steatosis accompanied by inflammation and hepatocellular injury (Fig. 3). It can further progress to fibrosis and cirrhosis [99]. For this review, studies labeled as NAFLD were included as MASLD, as they largely overlap with the current diagnostic criteria for MASLD. In this context, FGF21 and GDF15 have been implicated in different stages of MASLD/MASH and in the processes associated with hepatic fibrogenesis, highlighting their potential relevance across the spectrum of liver disease progression.

#### FGF21 and GDF15 in MASLD/MASH

Both FGF21 and GDF15 have emerged as key hepatokines whose hepatic expression and circulating concentrations are consistently elevated in MASLD [171–177] and further increase with disease progression, reflecting metabolic stress and hepatocellular injury [171, 175–180] (Fig. 3). In this context, GDF15 concentrations progressively increase during the sequential development of obesity, T2D and MASLD, with the liver emerging as a major source of GDF15 as MASLD progresses (Fig. 3) [141]. Accordingly, GDF15 has also been proposed as a potential non-invasive biomarker of liver disease progression (Fig. 3), as its circulating levels can predict fibrosis risk independently of classic metabolic confounders [180, 181], and hepatic *GDF15* expression predicts MASH more accurately than MASLD [141].

Mechanistically, FGF21 upregulation during MASLD appears to be peroxisome proliferator-activated receptor α (PPARα)-dependent [174, 182]. Notably, *Fgf21* deficiency increases susceptibility to MASH [183] and contributes to the progression from MASH to hepatocellular carcinoma [184]. Consistently, genetic and pharmacological studies have reported that FGF21 exerts hepatoprotective effects by reducing steatosis, inflammation and fibrosis while improving systemic metabolic homeostasis through enhanced lipid oxidation and reduced lipogenesis and inflammatory cell recruitment [34, 185–188], supporting its potential as a therapeutic target for MASLD [114, 189]. Importantly, plasma FGF21 concentrations negatively correlate with *Klb* expression in fatty liver conditions, suggesting possible hepatic FGF21 resistance during liver disease [174]. Experimental studies also support a protective role for GDF15 in liver injury, as its deficiency exacerbates steatosis, inflammation and fibrosis [138], whereas its overexpression promotes hepatic fatty acid β-oxidation and attenuates liver injury [109]. Overall, both exert protective effects by reducing steatosis, inflammation and fibrosis (Fig. 3), and their elevation appears to represent a compensatory response aimed at restoring metabolic homeostasis rather than a causal factor of disease progression. However, while FGF21 analogues are currently being studied in clinical trials for liver disease [190, 191], to our knowledge, there are no clinical trials yet evaluating GDF15 analogues in this context.

Interestingly, combined whole-body deletion of *Fgf21* and *Gdf15* worsens hepatic steatosis and insulin resistance compared with single *Gdf15* deletion. G*df15*-KO mice display increased hepatic and circulating FGF21 levels after prolonged HFD, whereas *Fgf21-*KO mice exhibit elevated circulating GDF15 concentrations [43], suggesting a compensatory crosstalk between the two hormones (Table 2). In a separate study, however, hepatic *Gdf15* overexpression in mice fed a HFD increased both hepatic *Fgf21* mRNA expression and circulating FGF21 concentrations, whereas hepatic *Fgf21* overexpression did not alter GDF15 levels, suggesting a unidirectional regulatory relationship (Table 2) [93]. Together, these findings suggest that the interaction between FGF21 and GDF15 is context-dependent and may vary according to the experimental model and the nature of the metabolic challenge. Complementing these findings, studies on mitochondrial dysfunction, an important contributor to MASLD development, support a complementary role of FGF21 and GDF15 in systemic metabolic adaptations [11]. Genetic models of mitochondrial stress, combined with *Fgf21* and/or *Gdf15* deletion, have shown that FGF21 primarily enhances insulin sensitivity, energy expenditure and thermoregulation [192–194], whereas GDF15 mainly regulates appetite, body weight and protection against diet-induced metabolic alterations [66, 192, 195], while also promoting energy expenditure in some contexts [196, 197]. Notably, simultaneous deletion of both *Fgf21* and *Gdf15* abolishes several adaptive metabolic responses [198], further supporting the notion of cooperative roles in mediating systemic responses.

Collectively, current evidence supports the concept that FGF21 and GDF15 are components of an endogenous adaptive response to hepatic metabolic stress rather than drivers of disease progression. However, the persistent elevation of both hepatokines despite ongoing liver injury suggests that their endogenous induction alone is insufficient to fully counteract hepatic metabolic stress. Whether this reflects inadequate compensatory responses, altered tissue sensitivity or other adaptive mechanisms remains to be elucidated.

#### FGF21 and GDF15 in liver fibrogenesis

**Fig. 5:**
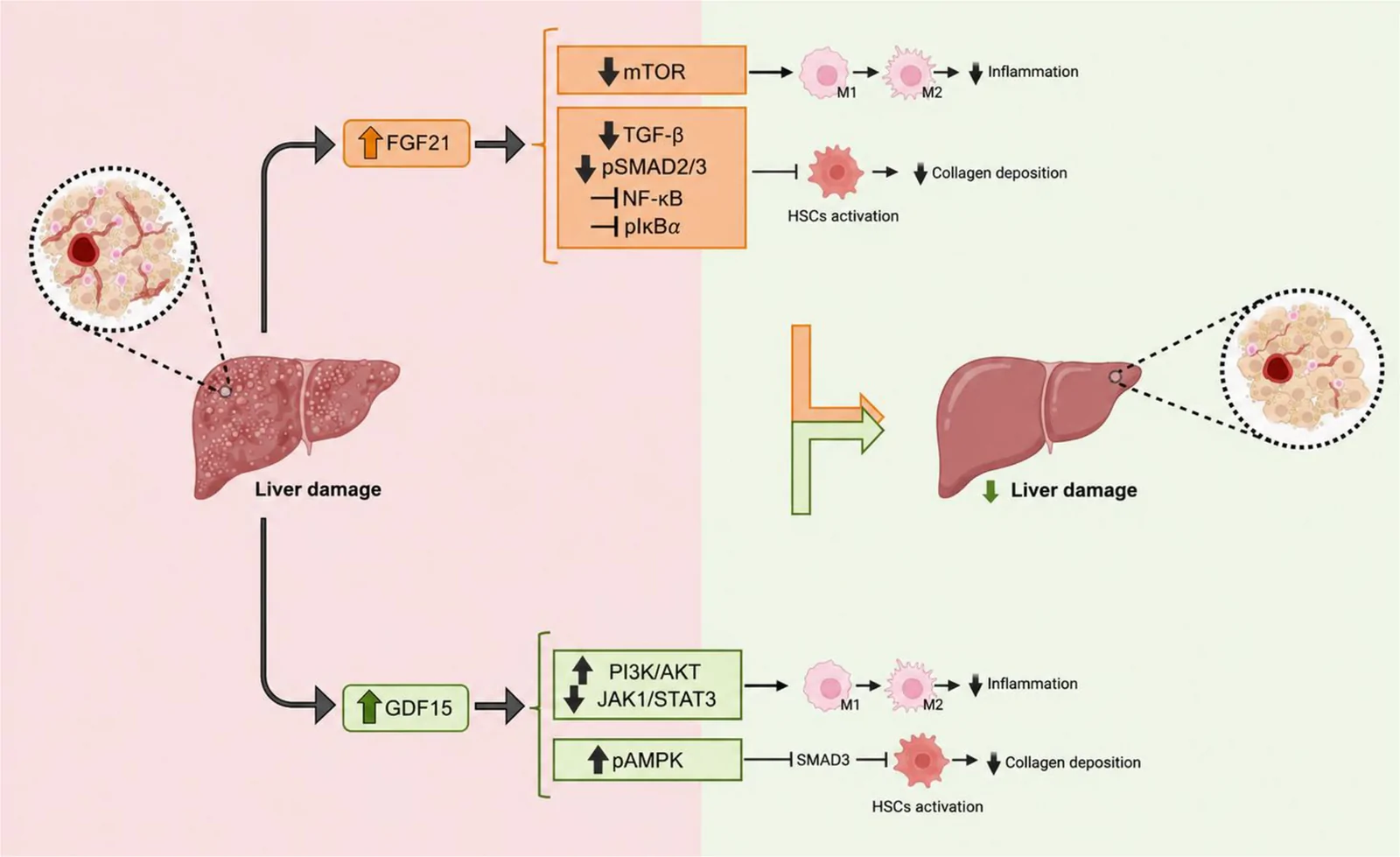
Main functions of FGF21 and GDF15 in liver injury. Liver injury induces *FGF21* and *GDF15* hepatic expression, increasing their circulating concentrations. Consequently, FGF21 and GDF15 activate signaling pathways that reduce inflammation, inhibit hepatic stellate cell activation decreasing collagen deposition, and limit fibrosis, ultimately alleviating liver damage. FGF21, fibroblast growth factor 21; GDF15, growth differentiation factor 15; mTOR, mechanistic target of rapamycin; TGF-β, transforming growth factor β; pSMAD2/3, phosphorylated mothers against decapentaplegic homolog 2/3; NF-κB, nuclear factor κB; pIκBα, phosphorylated inhibitor of κB α; PI3K, phosphoinositide 3-kinase; AKT, protein kinase B; JAK1, janus kinase 1; STAT3, signal transducer and activator of transcription 3; pAMPK, phosphorylated AMP-activated protein kinase, M1, pro-inflammatory macrophages; M2, anti-inflammatory macrophages; HSCs, hepatic stellate cells. Created using BioRender

Accumulating evidence regarding the predictive capacity of FGF21 and GDF15 for fibrosis development in the context of MASLD has increased the interest in elucidating their potential mechanistic involvement in the fibrogenic process [199, 200]. Fibrosis is the result of an excessive accumulation of extracellular matrix (ECM) components and impaired remodeling processes [201]. Several studies suggest that both molecules may not only serve as biomarkers but also modulate key pathways implicated in the activation of stellate cells, macrophage polarization, and extracellular matrix remodeling (Figs. 4, 5). In this context, FGF21 and GDF15 appear to counteract liver fibrosis through complementary anti-inflammatory and metabolic mechanisms. Both mitigate fibrogenesis by suppressing inflammation, inhibiting hepatic stellate cell (HSC) activation, reprogramming macrophage metabolism and modulating stress-related signaling pathways (Fig. 5).

FGF21 has been shown to exert anti-fibrotic effects through the reduction of inflammation [188]. Mouse models of diet-induced MASH lacking *Fgf21* exhibit increased hepatic expression of *Tgfb1* and elevated levels of inflammatory markers such as interleukin (IL)−1β, monocyte chemoattractant protein-1 (MCP-1) or cluster of differentiation 68 (CD68) [185], while liver *Fgf21* overexpression prevented inflammation, immune cell recruitment, and the activation of Kupffer cells, thereby inhibiting the activation of HSC, which are responsible for collagen production, ultimately alleviating liver fibrogenesis [188]. Mechanistically, FGF21 inhibits HSC activation by downregulating *Tgfb1* expression and mothers against decapentaplegic homolog 2/3 (SMAD2/3) phosphorylation, as well as by inhibiting nuclear factor κ-light-chain-enhancer of activated B cells (NF-κB) nuclear translocation and IκBα phosphorylation (Fig. 5) [202]. In mice with chronic pancreatitis, FGF21 treatment promoted macrophage polarization from a pro-inflammatory M1 phenotype toward an anti-inflammatory M2 phenotype via the mechanistic target of rapamycin (mTOR) signaling pathway, decreasing the secretion of pro-inflammatory cytokines such as IL-1β, tumor necrosis factor α (TNFα) and IL-6 (Fig. 5). This led to a reduction in pancreatic stellate cell activation and, therefore, to decreased collagen deposition [126]. In individuals with liver fibrosis, hepatic *Fgf21* mRNA and protein levels progressively increase with the severity of inflammation and fibrosis, while *Klb* expression decreases inversely. Treatment of Huh-7 cells with IL-1β upregulated FGF21 expression through the NF-κB and c-Jun N-terminal kinase (JNK) pathways [131].

Regarding GDF15, controversial data exist regarding its role in fibrosis. Jurado-Aguilar et al. [203] reported that GDF15 deficiency in mice impaired glucose metabolism and gluconeogenesis by reducing AMPK phosphorylation, while promoting hepatic fibrogenesis through activation of the TGFβ1/SMAD3 pathway. *Gdf15*-deficient mice also showed increased expression of pro-inflammatory macrophage gene markers such as *Tnfa* and *Mcp1* in the liver, further contributing to fibrosis. Their treatment with recombinant GDF15 restored AMPK, which in turn inhibited SMAD3 activity leading to improved glucose metabolism and reduced fibrotic gene expression (Fig. 5). Similarly, *Gdf15*-KO mice subjected to a chronic alcohol diet or chronic CCl_4_-induced liver injury exhibited exacerbated liver damage compared to WT mice, as evidenced by elevated circulating alanine aminotransferase/aspartate aminotransferase (ALT/AST) levels and upregulated inflammatory and fibrotic markers. Recombinant GDF15 treatment on them reduced inflammation, fibrosis and liver injury markers [138]. Consistently, in murine models of fibrosis induced by CCl_4_ or a 3,5-diethoxycarbonyl-1,4-dihydrocollidine (DDC) diet, *Gdf15* deletion enhanced fibrosis by leading to increased macrophage and neutrophil infiltration and elevated pro-inflammatory cytokines [139]. Conversely, GDF15 administration or hepatocyte-specific overexpression in both mouse models mitigated fibrosis through metabolic reprogramming of macrophages from the M1 to the M2 phenotype (Fig. 5), characterized by increased oxidative phosphorylation. This metabolic switch is associated with a suppression of the inflammatory response and reduced HSC activation [139]. Interestingly, despite these protective findings, Koo et al. [180] reported a pro-fibrotic role for GDF15, as treatment of human hepatic stellate cells (LX-2) with recombinant GDF15 treatment induced SMAD2/3 phosphorylation, thereby inducing fibrosis.

Collectively, the available evidence suggests that the anti-fibrotic actions of FGF21 and GDF15 are largely secondary to their ability to restore metabolic and inflammatory homeostasis rather than to directly target fibrogenic pathways. This suggests that modulation of metabolic stress, rather than direct inhibition of ECM production, may represent a key mechanism by which these stress-responsive hepatokines limit fibrosis progression. Moreover, their overlapping roles suggest a potential synergistic role in limiting fibrosis progression and preserving liver integrity (Fig. 3).

## Implications of FGF21 and GDF15 during weight loss and metabolic interventions

Lifestyle modification is generally the first approach to obesity management; however, the most effective treatment often requires a multimodal strategy. This approach typically combines diet modifications, increased physical activity, and pharmacological therapy. When these interventions are insufficient, bariatric surgery is considered as a subsequent therapeutic option [204]. Because FGF21 and GDF15 are stress- and nutrient-responsive endocrine mediators, weight loss interventions provide a physiological context in which changes in nutrient availability, energy balance and metabolic state can alter their circulating concentrations. Therefore, research over the past decades has focused on how different weight loss strategies affect their concentrations, driven by the interest in their potential as biomarkers and therapeutic targets for obesity and its associated complications.

### Effects of dietary interventions on FGF21 and GDF15

Changes in nutrient composition and availability can differentially influence FGF21 and GDF15, highlighting their sensitivity to distinct nutritional and metabolic signals. Consistent with the increase in FGF21 levels induced by high-carbohydrate and low-protein intake [48, 49, 59, 63], patients with T2D exhibited significantly reduced plasma FGF21 concentrations after 6 weeks of a carbohydrate-reduced high-protein diet [205]. This decrease occurred independently of weight loss and was not accompanied by alterations in GDF15 concentrations [205]. This suggests that changes in macronutrient composition may differentially regulate these mediators, with FGF21 appearing more responsive to such changes, whereas GDF15 may require different or more pronounced metabolic stress signals for its induction.

Fasting represents a different nutritional context characterized by nutrient deprivation, which leads to a progressive shift from glucose towards lipid utilization [206]. The decline in insulin and relative increase in glucagon during fasting promote WAT lipolysis, increasing the availability of circulating fatty acids, thereby promoting hepatic fatty acid oxidation and ketogenesis [207]. These metabolic changes are accompanied by alterations in FGF21 and GDF15 concentrations (Fig. 1), suggesting that both mediators may participate in the endocrine adaptation to the fasted state. However, FGF21 and GDF15 responses appear to depend on the duration of nutrient deprivation. While a decrease in FGF21 levels has been reported in humans during the early stages of fasting [208, 209], circulating FGF21 concentrations significantly increased after 7 days of fasting [209, 210]. Refeeding suppresses the induction of FGF21 after fasting [209, 211, 212]. Regarding GDF15, during 7 days of total fasting, its concentrations increased at day 3 and returned to baseline levels over time [108]. This temporal pattern may indicate that GDF15 responds to the acute metabolic stress associated with nutrient deprivation, whereas FGF21 may be more closely associated with longer-term metabolic adaptation. However, the evidence for GDF15 remains heterogeneous, as some studies have reported increased circulating GDF15 levels and hepatic *Gdf15* expression in mice subjected to fasting [213], whereas others found no significant changes in GDF15 levels but supported an elevation in FGF21 concentrations [108]. The increase in FGF21 levels during fasting occurs more rapidly in mice than in humans [209, 212] and is mediated, at least in part, by PPARα activation [210, 212], which is in turn promoted by the increased availability of fatty acids during fasting [212]. GDF15 induction during fasting has been linked to activation of the IRE1α-XBP1s branch of the unfolded protein response (UPR) in the liver [213]. In addition, emerging evidence suggests that, as observed for FGF21, increased fatty acid availability may contribute to GDF15 induction, although through stress-responsive pathways [214]. The PPARα-FGF21 axis may play a key role in maintaining glucose homeostasis under prolonged fasting conditions by promoting hepatic gluconeogenesis, an effect primarily mediated by activation of the hypothalamic–pituitary–adrenal (HPA) axis [211, 215]. However, Zhang et al. [213] did not find changes in the hepatic *Fgf21* expression after inhibiting *Ppara* expression, suggesting that other regulators may activate *Fgf21* expression as a compensatory mechanism for *Ppara* loss [212, 213]. The rise in FGF21 concentrations during fasting enhances WAT lipolysis and induces ketosis [211, 212]. Like FGF21, hepatic GDF15 upregulation during fasting has been shown to promote ketogenesis, while also enhancing fatty acid β-oxidation [213]. Therefore, despite differences in their mechanisms of induction and temporal regulation, the induction of FGF21 and GDF15 during fasting may contribute to a shared adaptive endocrine response that promotes the utilization of alternative energy substrates when glucose availability is limited.

Ketogenic diets are characterized by severe carbohydrate restriction and high fat intake, resulting in a metabolic shift from glucose utilization towards fatty acid oxidation and ketone body production [216]. This metabolic state is associated with increased circulating and hepatic FGF21 and GDF15 concentrations in both mice and humans (Fig. 1) [205, 217, 218]. GDF15 induction is mainly mediated via PPARγ-dependent transcriptional activation [218], whereas FGF21 induction is downstream of PPARγ and PPARα [211, 219]. Lu et al. showed that high levels of circulating GDF15 are essential for the reduction in food intake and the associated weight loss observed during ketogenic diet interventions, as these effects are markedly attenuated in *Gdf15*- or *Gfral*-deficient mice [218]. Notably, blocking *Fgf21* with a neutralizing antibody in *Gdf15-*deficient mice feed a ketogenic diet abolished the increased energy expenditure. These findings suggest that the metabolic effects of ketogenic diets involve a coordinated FGF21-GDF15 response in which the two mediators exert complementary effects, with GDF15 primarily reducing energy intake and FGF21 promoting energy expenditure [218]. Interestingly, although ketogenic diets increase *Fgf21* mRNA expression in the liver, and FGF21 improves insulin sensitivity, they have also been associated with hepatic insulin resistance [220]. This paradox may be explained by impaired FGF21 signaling in the liver, potentially due to reduced expression of FGF21 receptors after a ketogenic diet [221]. However, these effects may also be time-dependent, as mice fed a ketogenic diet for 8 weeks showed improved insulin sensitivity [222]. Interestingly, it has been shown that the metabolic effects attributed to ketogenic diets are primarily driven by their concomitant protein restriction rather than ketosis itself, since ketogenic diets with more than 5% of energy from protein did not increase hepatic *Fgf21* expression [223].

Overall, fasting and ketogenic diets promote a shift toward lipid utilization and ketogenesis and are associated with the induction of FGF21 and GDF15 through partially distinct nutritional and stress signals. FGF21 appears to be particularly sensitive to macronutrient composition and lipid availability, whereas GDF15 may be more closely associated with cellular stress. Nevertheless, their coordinated induction under conditions of nutrient deprivation or carbohydrate restriction suggests complementary roles in the adaptive response to altered nutrient availability.

### Effects of exercise on FGF21 and GDF15

Exercise represents a distinct physiological and metabolic stressor that transiently increases energy demand and challenges energy homeostasis across multiple tissues [224]. This physiological stress can induce endocrine responses that contribute to systemic metabolic adaptation, including changes in FGF21 and GDF15 concentrations (Fig. 1). Both GDF15 and FGF21 have been reported to increase during and after exercise [225, 226], although their responses differ in magnitude, kinetics and dependence on exercise characteristics. GDF15 concentrations increase after acute [227–229] and long-term exercise training [225, 230], with a further increase observed during the recovery period following acute exercise [230]. This response is attenuated following prolonged regular training, consistent with adaptation to repeated exercise exposure [230]. Accordingly, physically active people exhibit lower basal GDF15 concentrations than age-matched controls. However, intense endurance exercise still induces a marked but transient increase in GDF15 concentrations, indicating that its acute stress response is preserved despite lower basal concentrations [231]. In contrast, the effects of exercise on circulating FGF21 levels remain more heterogeneous [232–234]. Similarly to GDF15, habitually active individuals exhibit lower basal serum FGF21 concentrations than sedentary counterparts [235]. However, the FGF21 response appears to depend on modality and duration. Endurance, but not resistance, exercise induces a significant and sustained increase in plasma FGF21 concentrations in mice [236], while acute exercise also increases circulating FGF21 in humans [237]. Nevertheless, chronic training studies have reported both increases and decreases in circulating FGF21, depending on population and intervention characteristics [238]. Additionally, a study in marathon runners reported an increase in FGF21 and GDF15 concentrations following a race, with both returning to baseline levels within 48 h post-race. The rise in FGF21 levels was more pronounced compared to GDF15, with no correlation between them [225]. Together, these findings suggest that, although both mediators respond to exercise-induced metabolic stress, their circulating responses differ, with GDF15 showing a relatively robust acute stress response and FGF21 exhibiting greater context dependence, being influenced by exercise type, intensity, duration, and metabolic state.

The mechanisms underlying exercise-induced FGF21 and GDF15 expression are tissue-specific and involve both shared and distinct pathways. Exercise increases the glucagon-to-insulin ratio, inducing hepatic expression of both FGF21 and GDF15 [239, 240]. For FGF21, exercise-induced lipolysis increases fatty acid availability, enhancing PPARα and ATF4 signaling and promoting FGF21 expression [237]. In addition, exercise can increase hypothalamic FGF21 via central lactate uptake, which activates GPR81-p38-MAPK signaling, stimulating WAT lipolysis and fat mobilization [241]. GDF15 during exercise appears to involve stress-responsive pathways. Acute exercise increases GDF15 expression in skeletal muscle concomitantly with activation of UPR/ISR pathways [242]. However, evidence that canonical ER stress/CHOP signaling is dispensable for this response suggests that additional mechanisms contribute to its induction [243]. The potentially coordinated regulation of FGF21 and GDF15 is further supported by evidence that both mediators can be regulated by common post-transcriptional mechanisms. CNOT6L (CCR4-NOT transcription complex subunit 6-like) has been recently identified as a common post-transcriptional regulator of both FGF21 and GDF15 in response to metabolic stimuli such as fasting, exercise, and inflammation [244]. Inhibition of CNOT6L prevents their degradation, thereby elevating their circulating concentrations [244].

Exercise-induced changes in FGF21 and GDF15 may also contribute to metabolic adaptation. Exercise-induced increases in GDF15 have been associated with greater reductions in fat mass in individuals with obesity after long-term exercise, suggesting a potential role of GDF15 in exercise-induced metabolic adaptations [245]. However, exercise-induced increases in GDF15 do not appear to be required for the insulin-sensitizing effects of chronic exercise in lean or obese mice or in individuals with obesity [246]. Conversely, a decrease in FGF21 after a chronic endurance exercise program was associated with a reduction in hepatic fat content. Notably, exercise restores FGF21 sensitivity in WAT of DIO mice by upregulating FGFR1 and KLB expression via PPARγ activation, thereby improving insulin sensitivity and overall metabolic health [247].

The tissue sources of circulating GDF15 and FGF21 during exercise remain debated. For GDF15, one study reported a significant increase in *GDF15* mRNA levels in skeletal muscle after acute aerobic exercise, suggesting that skeletal muscle may contribute to circulating GDF15 concentrations [248]. GDF15 released from skeletal muscle appears to target WAT to promote lipolysis [248], which could explain the association between increased GDF15 levels and the reduction in fat mass induced by exercise [245]. However, several studies found no significant contribution of skeletal muscle to circulating GDF15 levels after exercise [231, 240, 249, 250], instead suggesting that the liver is the major source of exercise-induced GDF15 [240, 243]. For FGF21, the liver also appears to be the main source of circulating FGF21 levels after exercise, as acute exercise increases hepatic *Fgf21* expression but not its expression in skeletal muscle or BAT [237, 251]. However, high-intensity running has been shown to induce *Fgf21* expression in skeletal muscle in mice, suggesting that muscle-derived FGF21 expression might be induced in an intensity-dependent manner [252]. Therefore, the liver appears to be the main source of circulating FGF21 and GDF15 concentrations during exercise, although skeletal muscle may contribute under specific exercise conditions.

Collectively, current evidence suggests that both FGF21 and GDF15 participate in the systemic adaptations to physical activity, although their regulation, kinetics and tissue sources may differ. Acute exercise generally induces a transient increase in circulating FGF21 and GDF15 concentrations, whereas regular training is associated with lower basal concentrations, suggesting that these factors are more indicative of metabolic stress than of physical activity itself. From this perspective, regular exercise may attenuate the chronic activation of stress-response pathways associated with obesity and aging while preserving their adaptive activation in response to acute physiological challenges. Nevertheless, whether FGF21 and GDF15 are essential mediators of the beneficial metabolic effects of exercise or primarily serve as biomarkers of the adaptive response remains an important unresolved question.

### Effects of bariatric surgery on FGF21 and GDF15

Bariatric surgery is considered one of the most effective interventions for weight loss. Body weight reduction is primarily achieved through decreased food intake and absorption, in combination with alterations in food preferences, and broader metabolic adaptations [253]. Beyond weight loss itself, bariatric surgery induces profound metabolic remodeling by simultaneously altering nutrient availability, gastrointestinal signaling, bile acid metabolism and energy homeostasis [254]. These changes are accompanied by alterations in circulating FGF21 and GDF15 concentrations, suggesting that both mediators may participate in the endocrine response to the metabolic remodeling induced by bariatric surgery.

The postoperative regulation of FGF21 and GDF15 is highly heterogeneous, with responses depending on the surgical procedure, follow-up period, and baseline metabolic phenotype. Regarding FGF21, some studies show an increase in FGF21 levels after bariatric surgery [255–257], whereas others have not observed significant differences [258], or even a decrease in its levels [258–260]. In this sense, differences between surgical procedures have been reported. While some authors have observed that plasma FGF21 concentrations increase after Roux-en-Y gastric bypass (RYGB) surgery but not after laparoscopic adjustable gastric banding (LAGB) surgery [261], others have found that FGF21 levels remained unchanged after RYGB but decreased after sleeve gastrectomy (SG) surgery [258]. Moreover, a meta-analysis showed that an acute increase in FGF21 concentrations was no longer evident 18 months after surgery, suggesting that the FGF21 response is influenced by the postoperative metabolic adaptation [262]. These temporal changes may be biologically relevant, as a greater increase in circulating FGF21 levels 3 months after bariatric surgery has been associated with greater weight loss one year after surgery, suggesting that the early plasma FGF21 response after surgery may serve as a predictor of subsequent weight loss [257]. However, elevated preoperative FGF21 concentrations in our cohort were associated with greater difficulty losing excess weight and fat mass after RYGB [260].

Inconsistent evidence has also been observed in relation to GDF15. While some authors have shown an increase in GDF15 levels after RYGB [260, 263, 264] and SG [265, 266], others have observed a decrease in GDF15 concentrations after RYGB and SG [259, 267]. The significant increase in circulating GDF15 levels after SG and RYGB has usually been related to the degree of weight loss [264–266]. However, decreased concentrations of both GDF15 and FGF21 after bariatric surgery have also been associated with greater weight loss at one year [259] as well as with metabolic improvements [267]. Moreover, like FGF21, GDF15 concentrations after bariatric surgery also appear to be influenced by the follow-up period, with GDF15 levels increasing in the months following bariatric surgery and returning to baseline levels over time [260]. Therefore, the direction of change alone may not capture the biological significance of these mediators, but their temporal pattern may reflect the transition from acute metabolic stress to longer-term metabolic adaptation.

The mechanisms underlying FGF21 and GDF15 changes after bariatric surgery are not well understood. The increase in FGF21 may be related to changes in bile acid metabolism. Bariatric procedures, particularly RYGB, increase circulating bile acid levels, which may contribute to the upregulation of hepatic *Fgf21* expression and secretion through intestinal FGF19/15 signaling [268]. In contrast, the mechanisms responsible for the increase in GDF15 after bariatric surgery remain largely unknown. Notably, although increases in circulating FGF21 and GDF15 concentrations after bariatric surgery may contribute to the reduced food intake and weight loss induced by surgery, whether these changes are essential for these improvements remains to be elucidated. Indeed, studies in mice suggest that FGF21 and GDF15 signaling is not essential for metabolic improvements after bariatric surgery [253, 269], although FGF21 may play a minor role in food choice and locomotor activity [269].

Overall, despite the heterogeneity of findings, both proteins show potential as biomarkers for monitoring and predicting postoperative weight loss and metabolic outcomes. Early increases in FGF21 and GDF15 may reflect responses to acute metabolic stress induced by surgery, whereas longer-term changes may reflect the progressive metabolic adaptation and resolution of obesity-related metabolic dysfunction. However, further studies are required to determine whether changes in their circulating levels contribute to the beneficial effects of bariatric surgery or primarily reflect the underlying metabolic remodeling.

### Effects of metformin on FGF21 and GDF15

Metformin is a widely prescribed medication for T2D due to its glucose-lowering effects and can also induce weight loss through appetite suppression [270]. Metformin treatment has been shown to increase circulating FGF21 and GDF15 levels in both rodents and humans (Fig. 1) [271–277], with several studies reporting increases in *FGF21* mRNA and protein expression in the liver, skeletal muscle and WAT [271, 278]. However, other studies have reported reduced circulating FGF21 levels after metformin treatment, suggesting that these responses may depend on treatment duration or metabolic context [279]. Metformin also enhances the mRNA and protein expression of KLB and FGFR1c in WAT, thereby improving FGF21 signaling [278]. Interestingly, Day et al. [274] demonstrated that GDF15 was one of the most strongly upregulated genes in primary mouse adipocytes after metformin exposure, suggesting that GDF15 may contribute to the beneficial effects of metformin on weight loss [274, 275]. Consistent with a potential role of GDF15 in weight regulation, children with obesity treated with metformin showed increased serum GDF15 concentrations, and a greater increase in GDF15 was associated with greater loss of body weight and visceral fat [277]. However, Klein et al. [276] reported that the GDF15-GFRAL pathway was not essential for the effects of metformin on body weight, suggesting that the contribution of GDF15 to metformin-induced weight loss may be context-dependent.

Mechanistically, the induction of FGF21 and GDF15 has been associated with stress-related pathways. Regarding FGF21, some studies suggest that metformin-induced FGF21 expression is mediated by AMPK activation [280], whereas others have reported that metformin increases hepatic *Fgf21* expression in mice through ATF4, triggered by activation of the ISR, but independently of AMPK, mTORC1 and PPARα [273]. Similarly, GDF15 induction has been associated with ATF4 and CHOP, although the role of AMPK remains debated, with evidence supporting both AMPK-dependent and -independent mechanisms depending on the experimental model [119, 274, 281]. Additionally, metformin reverses the hyperglycemia-induced decrease in FGF21 expression and AKT phosphorylation in pancreatic islets from diabetic rats, both in vivo and in vitro, suggesting that its beneficial actions on insulin synthesis and secretion may be mediated, at least in part, through the FGF21/AKT signaling pathway [282].

Taken together, these findings suggest that FGF21 and GDF15 may represent complementary components of the adaptive endocrine response to metformin. Although both mediators can be induced through stress-responsive pathways, their distinct regulation and downstream actions may account for their differential contributions to metabolic homeostasis and body weight regulation during metformin treatment.

## FGF21 and GDF15 in aging

**Fig. 6:**
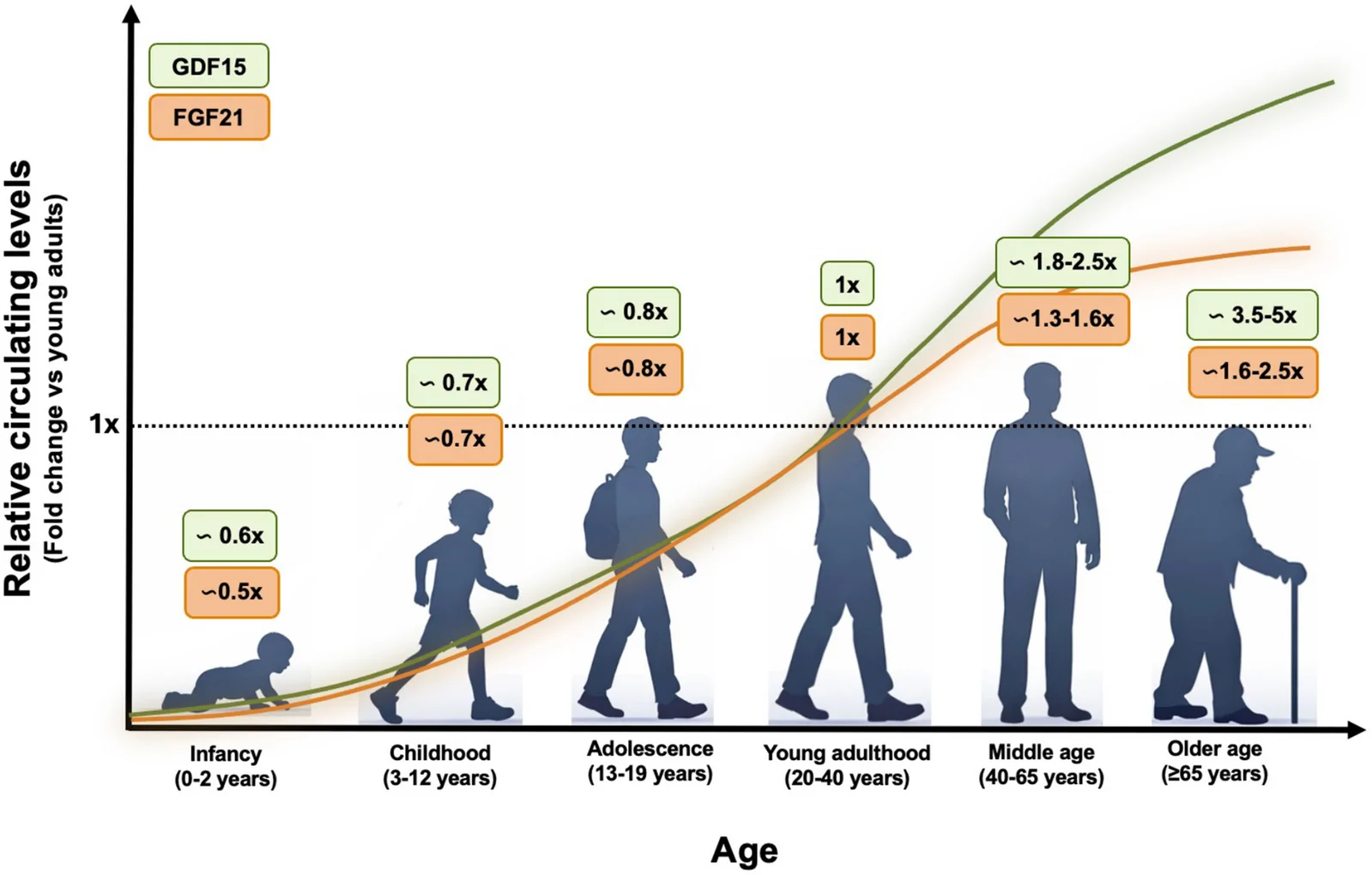
Schematic representation of age-related changes in circulating FGF21 and GDF15 concentrations during healthy aging. Both GDF15 and FGF21 tend to increase with advancing age, with GDF15 exhibiting a pronounced age-related increase while FGF21 shows a moderate increase. The curves represent approximate relative changes in circulating levels throughout the lifespan, normalized to young adulthood, showing general trends observed in the literature rather than absolute circulating concentrations, which may vary substantially according to population characteristics and analytical methods. FGF21, fibroblast growth factor 21; GDF15, growth differentiation factor 15. Created using BioRender

Aging is a major risk factor for numerous pathologies, as it is characterized by a progressive decline in cellular function and the deterioration of multiple tissues, increasing vulnerability to disease and mortality [283]. This process is driven by the progressive accumulation of cellular and molecular damage and involves twelve interconnected hallmarks, categorized into primary, antagonistic and integrative hallmarks [7]. Primary hallmarks initiate cellular damage (genomic instability, telomere attrition, epigenetic alterations, loss of proteostasis and disabled macroautophagy); antagonistic hallmarks involve compensatory responses that, when chronically activated, can become deleterious (deregulated nutrient sensing, mitochondrial dysfunction, cellular senescence); and integrative hallmarks ultimately contribute to tissue and organ decline (stem cell exhaustion, altered intercellular communication, dysbiosis and chronic inflammation) [7, 284]. With advancing age, the accumulation of cellular damage progressively disrupts cellular homeostasis and increases cellular stress, contributing to progressive tissue dysfunction. In response to these age-related stressors, compensatory pathways are activated to preserve cellular and metabolic homeostasis. Circulating FGF21 and GDF15 concentrations have been shown to increase with aging (Figs. 3, 6), suggesting that both mediators may contribute to systemic adaptation to age-related cellular dysfunction.

Although there is limited evidence linking FGF21 and GDF15 with primary hallmarks of aging, both have been associated with several antagonistic and integrative hallmarks. Mitochondrial dysfunction represents an important source of cellular stress during aging and contributes to the induction of both FGF21 and GDF15 [285, 286]. Both mediators have been proposed to act as endocrine signals of mitochondrial stress, linking local cellular dysfunction to systemic metabolic adaptation. Experimental studies support a context-dependent role of FGF21 and GDF15 in response to mitochondrial stress, depending on the severity of the stress [287]. For example, adeno-associated viral (AAV)-mediated FGF21 gene therapy in aged mice enhanced mitochondrial function [288]. However, under severe mitochondrial stress, FGF21 may exert detrimental effects*. Opa1* deletion in adult mice increased muscle *Fgf21* expression and circulating FGF21 concentrations, leading to premature death, which was rescued by simultaneous *Fgf21* deletion [289], whereas FGF21 elevation following *Opa1* deletion in skeletal muscle prevented age- and diet-induced weight gain and insulin resistance [193]. Similarly, *GDF15* depletion in human dermal fibroblasts induces mitochondrial dysfunction and premature senescence [290], supporting a physiological role in maintaining cellular homeostasis during aging. Together, these findings suggest that both FGF21 and GDF15 may act as adaptive signals in response to mitochondrial dysfunction in aging, although their effects appear to depend on the severity of the underlying stress.

Cellular senescence and chronic inflammation are also related to FGF21 and GDF15 signaling. Aging is associated with an accumulation of pro-inflammatory macrophages, and FGF21 administration has been shown to reduce liver injury and senescence markers during aging by promoting macrophage polarization from the M1 to M2 phenotype, potentially via AMPKα activation and NF-κB inhibition [127]. These findings align with studies linking FGF21 to AMPK activation, improving metabolic health and leading to lifespan extension [291]. Nevertheless, FGF21 has also been reported to extend lifespan independently of AMPK [292], suggesting that its effects are mediated through both AMPK-dependent and -independent mechanisms. Moreover, FGF21/KLB signaling to adipocytes has been shown to be essential for the anti-senescence effects associated with protein restriction [293]. Similarly, GDF15 has been reported to play an important role in attenuating age-related inflammation in the liver and WAT [135]. Interestingly, metformin treatment promotes M2 macrophage polarization in the liver, similarly to FGF21 administration [127]. As metformin increases GDF15, and GDF15 has been associated with macrophage polarization toward the M2 phenotype [139], these findings suggest that both FGF21 and GDF15 may contribute to preserving liver function during aging. Importantly, GDF15 is considered a component of the senescence-associated secretory phenotype (SASP) [294], linking its induction during aging with cellular senescence, and in chronic inflammatory states it has been shown to act as an immunosuppressive factor, promoting aging [295].

Interestingly, experimental studies support a context-dependent role of FGF21 in aging. Hepatocyte- and adipocyte-specific *Fgf21* overexpression in mice increased circulating FGF21 concentrations, improved metabolic health and extended lifespan. However, reduced bone mass has been reported following hepatocyte-specific *Fgf21* overexpression [292, 296]. Conversely, older *Fgf21*-KO mice developed lung inflammation, elevated inflammatory cytokines, hepatic steatosis, and metabolic alterations, whereas these abnormalities were absent in young *Fgf21*-KO mice, suggesting that FGF21 exerts a protective role that becomes more critical during aging [297]. Consistent with this, increased *KLB* expression has been reported in scWAT of older adults compared with young adults, suggesting that elevated circulating FGF21 concentrations with age do not result from reduced FGF21 responsiveness [298].

Notably, elevated GDF15 levels reduce appetite, a feature linked to aging, suggesting that its chronic elevation may contribute to the anorexia of aging and the associated functional decline, and positioning GDF15 as an interesting candidate to combat the age-associated impairments [299]. Like FGF21, GDF15 appears to exert beneficial effects under certain conditions, although its chronic elevation during aging may also have detrimental consequences for the aging process [28].

Interestingly, despite preclinical studies showing the potential of FGF21 and GDF15 as therapeutic targets for age-related decline, higher circulating concentrations of both proteins have been associated with reduced survival in older individuals [300, 301], and have been proposed as promising biomarkers of frailty and predictors of adverse outcomes in older adults [302, 303]. GDF15, in particular, has shown stronger associations with aging [146, 300], frailty [303] and mortality [300] than FGF21 (Fig. 3).

Importantly, chronological age, determined by the time elapsed since birth, does not necessarily correspond to biological aging, which reflects the cumulative decline in physiological function and health status [304]. In this context, FGF21 and GDF15 have been proposed as potential biomarkers of biological aging. However, circulating FGF21 and GDF15 concentrations have also been shown to increase with chronological aging (Fig. 6), as increased levels of both mediators with aging have been reported to be independent of health status [300, 305, 306] and physical activity levels [231]. In line with this, several studies have identified GDF15 as one of the most upregulated proteins during aging [300, 305, 307], supporting its potential value as a biomarker of chronological aging [308]. Moreover, GDF15 has been shown to be associated with primary hallmarks of aging. Negative correlations have been reported between circulating GDF15 levels and telomere length and telomerase activity, further supporting its role as a potential biomarker of aging [308, 309].

Despite their biomarker potential, it remains unclear whether their elevated levels exert beneficial or detrimental effects. While experimental models support beneficial roles in maintaining metabolic homeostasis and delaying age-related dysfunction, their chronic elevation during aging is associated with frailty, reduced longevity and mortality. These findings suggest that increased circulating FGF21 and GDF15 concentrations may represent adaptive stress responses that become insufficient or maladaptive during advanced aging, potentially leading to detrimental effects. Alternatively, their elevation may primarily reflect cumulative cellular stress and biological aging rather than directly mediate age-related decline.

## FGF21 and GDF15 in the emerging interplay between metabolic disorders and aging

As previously discussed, the relationship between metabolic diseases and aging is driven by shared underlying mechanisms, including mitochondrial dysfunction, ER and oxidative stress, and chronic low-grade inflammation (Fig. 1). These processes converge to disrupt metabolic homeostasis, impair tissue function, and accelerate systemic decline. Importantly, aging and metabolic dysfunction are tightly interconnected processes. The progressive decline in metabolic function with age disrupts metabolic homeostasis, while impaired metabolic homeostasis in turn accelerates biological aging [7, 310]. Thus, metabolic diseases and aging may form a self-reinforcing cycle in which chronic cellular stress progressively compromises tissue function and further impairs metabolic homeostasis. Within this context, FGF21 and GDF15 may represent components of a shared endocrine stress response that counteract the systemic decline associated with metabolic disorders and aging, modulating cellular stress and chronic inflammation. Interestingly, evidence that FGF21 responsiveness is not impaired in WAT during aging suggests that the age-related increase in FGF21 levels during aging [298] may reflect a distinct regulation of FGF21 signaling compared with metabolic diseases, in which, as previously described, FGF21 responsiveness is reduced [311]. Further studies should determine the mechanisms underlying these differences and their functional relevance to metabolic adaptation during aging.

Among the tissues most profoundly affected by aging is WAT, which undergoes substantial remodeling, mainly characterized by redistribution of fat depots, reduced lipid storage capacity, increased cellular senescence, and enhanced secretion of pro-inflammatory mediators, exhibiting several features of obesity-associated WAT dysfunction [312–315]. These alterations contribute to chronic low-grade inflammation and metabolic dysfunction. Accordingly, the age-associated increase in circulating FGF21 and GDF15 concentrations may initially represent an adaptive response aimed at counteracting tissue stress and preserving systemic metabolic homeostasis.

Evidence that FGF21 may contribute to the interplay between metabolic homeostasis and aging comes from experimental models in which metabolic improvements mediated by FGF21 are associated with increased lifespan. Indeed, the lifespan-extending effects of FGF21 have been proposed to be mediated, at least in part, by its beneficial effects on metabolic health [18]. One potential mechanism underlying this effect involves the activation of AMPK. As previously mentioned, FGF21 activates AMPK signaling, and thereby contributes to the regulation of glucose uptake, suggesting that the FGF21-AMPK axis may represent an important link between metabolic regulation and longevity [35, 36, 291]. In line with this, adipocyte-specific FGF21 overexpression improved metabolic health and extended lifespan in mice fed a HFD [296]. Further support for a role of FGF21 in longevity through metabolic effects comes from dietary protein restriction, which has been associated with increased lifespan and with increased FGF21 levels. Importantly, FGF21 has been shown to be essential for the lifespan-extending effects of protein restriction, by improving metabolic health [316–318]. Consistently, intramuscular treatment of geriatric mice with AAV-FGF21 also promoted healthspan extension by improving metabolic health, muscle function, and cognition. Specifically, the treatment was associated with enhanced mitochondrial function, restored proteostasis, and reversal of inflammation and fibrosis, as well as with activation of the AMPK signaling pathway [288]. Together, these findings support the potential contribution of FGF21 to healthy aging partly through promoting metabolic homeostasis. In contrast, despite the described beneficial effects of GDF15 on metabolism, evidence linking GDF15 to longevity through metabolic improvements is more limited. *Gdf15* overexpression in mice has been shown to increase lifespan, an effect associated with improvements in metabolism, including increased energy expenditure and downregulation of insulin/IGF-1/mTOR signaling [319]. However, this potential relationship remains poorly understood, raising the possibility that GDF15 may influence aging through the modulation of cellular stress. Further studies are needed to determine whether GDF15, like FGF21, may contribute to healthy aging through its effects on metabolic homeostasis.

Beyond metabolism and aging, elevated circulating levels of FGF21 and GDF15 have also been observed in a wide spectrum of diseases in which chronic inflammation and cellular stress play pivotal roles, including cardiovascular [29, 320, 321] and neurodegenerative diseases [322–327], sarcopenia [328–330], arthropathies [331, 332], renal dysfunction [333–335], and cancer [336–340]. Notably, the development of these conditions is commonly associated with aging. Interestingly, while in some contexts increased FGF21 and GDF15 levels have been linked to beneficial adaptive responses, in several conditions their elevation also correlates with disease severity and mortality. This apparent discrepancy may reflect a scenario in which these factors are upregulated in an attempt to counteract cellular stress, but when the stress is chronic, the compensatory response is insufficient, and high levels become markers of poor prognosis. This broad responsiveness supports their potential utility as integrated biomarkers of cellular distress and systemic adaptation, while also raising important questions about their context-dependent effects, ranging from protective to maladaptive depending on disease stage, tissue specificity, and the chronicity of their activation (Fig. 1).

Collectively, these observations support the concept that metabolic diseases and aging are interconnected processes driven by shared mechanisms of cellular stress. Within this context, FGF21 and GDF15 may act as components of a coordinated endocrine response to cellular stress improving metabolic dysfunction and aging. However, their potential contributions to this interplay may not be equivalent. Experimental evidence suggests that FGF21 may promote a healthy lifespan, at least in part through improvements in metabolic health, representing a potential target to simultaneously address metabolic disorders and age-related decline. GDF15 may similarly contribute to healthy aging through its effects on energy balance, although the extent to which these effects translate into increased lifespan remains less well established. Although evidence for potential interactions between FGF21 and GDF15 is emerging in the metabolic context, much less is known about whether and how these mediators may interact during aging. Future studies should clarify whether FGF21 and GDF15 act independently or through coordinated pathways during aging.

## Translational research and clinical implications of FGF21 and GDF15

As discussed throughout this review, both FGF21 and GDF15 have been associated with disease severity and clinical outcomes across metabolic disorders, as well as frailty and mortality, suggesting that their circulating concentrations may provide information on disease status. However, as they are elevated in numerous pathological contexts, increased concentrations may reflect the stress response associated with these disorders rather than a specific disease signal. Therefore, the lack of disease specificity may limit their utility as diagnostic biomarkers. Their combined assessment with other established biomarkers may provide complementary information and potentially improve their diagnostic value in metabolic diseases. Moreover, their dynamic regulation in response to metabolic interventions raises the possibility of using FGF21 and GDF15 to monitor treatment responses or as prognostic biomarkers. Changes in their circulating concentrations following bariatric surgery and lifestyle or pharmacological interventions may provide complementary information on the biological response to treatment, although whether these changes reliably predict long-term clinical benefit remains to be established. Importantly, prospective studies are needed to determine whether circulating FGF21 and GDF15 concentrations can improve patient stratification beyond conventional clinical and metabolic parameters. Therefore, whether specific FGF21-GDF15 profiles can distinguish disease states, predict treatment response, or identify patients who may benefit from specific treatments remains an important area for future investigation.

### Therapeutic potential and clinical development of FGF21- and GDF15-based strategies

Preclinical studies have supported the development of FGF21 and GDF15 as therapeutic targets for obesity and metabolic diseases (Table 3). In animal models, FGF21 consistently improves glucose homeostasis, lipid metabolism, hepatic steatosis and body weight, whereas GDF15 primarily reduces food intake and body weight (Table 3). Clinical translation has been more successful for FGF21, particularly in MASLD/MASH, where several analogues have consistently demonstrated the ability to reduce hepatic steatosis and fibrosis and to improve metabolic dysfunction, although their effects on body weight and glycemic control remain modest (Table 4). Reflecting this progress, three FGF21 analogues, efruxifermin, pegozafermin and efimosfermin alfa, are currently being evaluated in phase III trials for MASH with fibrosis (Table 4). Moreover, current clinical research is also expanding beyond FGF21 monotherapy. Combination strategies with incretin-based therapies, such as glucagon-like peptide-1 (GLP-1) receptor agonists, are being investigated in preclinical [341–345] and clinical studies [346, 347] to determine whether their complementary metabolic and hepatic effects can improve outcomes in obesity, T2D and MASLD.

**Table 3:** Representative preclinical studies supporting FGF21 and GDF15-based therapies

Intervention	Disease	Model	Approach	Main effects	Main adverse effects	Reference
**FGF21-based therapies**						
Recombinant FGF21	Obesity	DIO mice	Recombinant murine FGF21	Reduced body weight and fat mass Increased energy expenditure Improved glycemic control Improved lipid metabolism Reversed hepatic steatosis	No major safety signals reported	Jing et al. (2009) [33]
	Obesity	Obese rhesus macaque	Native recombinant human FGF21	Reduced body weight and fat mass, without affecting food intake Improved insulin sensitivity and reduced triglycerides	No major safety signals reported	Andersen et al. (2018) [348]
	Obesity/T2D	*ob/ob* and *db/db* mice	Recombinant human FGF21	Improved glycemic control and reduced triglycerides	No major safety signals reported	Kharitonenkov et al. (2005) [35]
	Obesity/T2D	New Zealand Obese mice (mice model of polygenic obesity and T2D)	Recombinant human FGF21	Prevented the onset of diabetes Improved glucose clearance Increased energy expenditure	Increased food intake	Laeger et al. (2018) [349]
	MASH	Diet-induced MASH mice	Recombinant human FGF21	Reduced hepatic steatosis and fibrosis Reduced hepatic cholesterol	No major safety signals reported	Rose et al. (2025) [187]
LY2405319	T2D	Diabetic monkeys	FGF21 variant	Reduced body weight and food intake	No major safety signals reported	Adams et al. (2013) [350]
Pegozafermin	T2D	Diabetic monkeys	GlycoPEGylated FGF21 analogue	Reduced body weight and food intake Improved dyslipidaemia and glycemic profile Increased adiponectin	No major safety signals reported	Rosenstock et al. (2023) [351]
PF-05231023	Obesity	Obese monkeys	Long acting FGF21 analogue	Reduced body weight, food intake and triglycerides Increased adiponectin	No major safety signals reported	Talukdar et al. (2016) [352]
	MASH	GAN DIO-MASH mice		Reduced body weight Reduced hepatic steatosis and fibrosis	No major safety signals reported	Nielsen et al. (2023) [353]
**GDF15-based therapies**						
Recombinant GDF15	Obesity	DIO mice and spontaneous obese cynomolgus monkeys	Recombinant GDF15	Reduced food intake and body weight	No major safety signals reported	Mullican et al. (2017) [25]
	Obesity	DIO mice and rats	Recombinant human GDF15	Increased insulin action independent of weight loss	No major safety signals reported	Sjøberg et al. (2023) [41]
LY3463251	Obesity	DIO mice, rats and spontaneous obese monkeys	GDF15/GFRAL agonist	Reduced food intake and body weight	No major safety signals reported	Benichou et al (2023) [354]
MBL949	Obesity	DIO mice, obese cynomolgus monkeys	Long-acting GDF15 analogue	Reduced food intake, body weight and adiposity Improved glucose metabolism	Generally safe and well tolerated No major safety signals reported	Smith et al (2025) [355]

*FGF21* fibroblast growth factor 21, *T2D* type 2 diabetes, *MASH* metabolic dysfunction-associated steatohepatitis, *DIO* diet-induced obesity, *ob/ob* leptin-deficient mice, *db/db* leptin receptor-deficient mice, *GAN* gubra amylin MASH, *GDF15* growth differentiation factor 15, *GFRAL* glial cell-derived neurotrophic factor family receptor α-like

**Table 4:** Clinical development of representative FGF21 and GDF15-based therapies in metabolic diseases

Drug	Disease	Approach	Phase	Main effects	Main adverse effects	Status	Reference
**FGF21-based therapies**							
LY2405319	Obesity, T2D	FGF21 variant Subcutaneous daily injections	Phase I	Reduced body weight and fasting insulin levels Improvements in dyslipidaemia	No serious adverse events	Completed	Gaich et al. (2013) [356] NCT01869959
PF-05231023	Obesity, T2D	Long acting FGF21 analogue	Phase I	Reduced body weight and triglycerides Increased adiponectin and IGF1	Nausea, vomiting, diarrhea	Completed	Talukdar et al. (2016) [352] NCT01396187
Pegbelfermin (BMS-986036)	Obesity, T2D	PEGylated FGF21 analogue	Phase IIa	Improved liver-related biomarkers in patients at risk of developing MASH Increased adiponectin	Injection-site bruising and diarrhea	Completed	Charles et al. (2019) [357] NCT02097277
	MASH		Phase IIa	Decreased hepatic steatosis Improved markers of hepatic injury and fibrosis and lipid profile Increased adiponectin	Nausea and diarrhea	Completed	Sanyal et al. (2019) [358] NCT02413372
	MASH + Fibrosis F3		Phase IIb	Reduced hepatic steatosis Improved liver injury, inflammation and fibrosis	Nausea and diarrhea	Completed	Loomba et al. (2024) [359] NCT03486899
	MASH + Fibrosis F4		Phase IIb	Improved non-invasive imaging and biomarker endpoints suggesting reduced disease activity and fibrosis	Nausea and diarrhea	Completed	Abdelmalek et al. (2024) [360] NCT03486912
Pegozafermin	MASLD with high risk of MASH/MASH + Fibrosis F1-F3	GlycoPEGylated FGF21 analogue	Phase Ib/IIa	Reduced hepatic steatosis, circulating lipids and fibrosis markers	Increased appetite (non-weight regain)	Completed	Loomba et al. (2023) [361] NCT04048135
	MASH + Fibrosis F2/F3		Phase IIb	Reduced hepatic steatosis and fibrosis	Nausea and diarrhea	Completed	Loomba et al. (2023) [362] NCT04929483
	MASH + Fibrosis		Phase III	-	-	Recruiting	NCT06318169
Efimosfermin alfa (BOS-580)	Obesity, MASH	Long-acting FGF21 analogue	Phase IIa	Reduced hepatic steatosis and fibrosis Reduced inflammation Reduced Hb1Ac Mild weight reduction	Nausea, vomiting, diarrhea	Completed	Loomba et al. (2025) [363] NCT04880031
	MASH + Fibrosis F4		Phase II	-	-	Recruiting	NCT06920043
	MASH + Fibrosis F2/F3		Phase III	-	-	Recruiting	NCT07221227 NCT07221188
Efruxifermin (AKR-001)	MASH	Long-acting Fc-FGF21 fusion protein	Phase IIa	Reduced hepatic steatosis and liver injury markers	Nausea, vomiting and diarrhea	Completed	Harrison et al. (2021) [364] NCT03976401
	MASH + Fibrosis F2/F3		Phase IIb	Fibrosis regression and and resolved steatohepatitis Enhanced insulin sensitivity and glycemic control Improved lipid profile Modest weight loss	Nausea and diarrhea	Completed	Harrison et al. (2023) [365] NCT04767529
	MASH + compensated cirrhosis F4		Phase IIb	Reduced fibrosis but not at 36 weeks	Nausea, diarrhea, increased appetite and administration-site reactions	Completed	Noureddin et al. (2025) [366] NCT05039450
	MASH + Fibrosis		Phase III	-	-	Recruiting	NCT06215716
BFKB8488A	Obesity	FGF21/KLB agonist	Phase I	Reduced body weight Improved lipid metabolism and cardiometabolic parameters Increased adiponectin	Nausea	Completed	Baruch et al. (2020) [367] NCT02593331
	T2D/MASLD		Phase Ib	Reduced hepatic steatosis Improved lipid metabolism Increased adiponectin	Nausea, vomiting, diarrhea and decreased appetite	Completed	Wong et al. (2023) [368] NCT03060538
**GDF15-based therapies**							
AMG-171	Obesity	Long-acting GDF15 analogue	Phase I	Non-published data	Non-published data	Terminated The totality of the data did not support continuation of its development	Non-published NCT04199351
LY3463251	Obesity	GDF15/GFRAL agonist	Phase I	Reduced food intake with a modest effect on body weight	Nausea, vomiting, abdominal pain and diarrhoea; dose-dependent	Terminated Insufficient benefit/tolerability ratio	Benichou et al. (2023) [354] NCT03764774
MBL949	Obesity	Long-acting acylated GDF15 analogue	Phase I-II	Non-relevant weight loss	Nausea	Terminated Insufficient benefit/tolerability ratio	Smith et al. (2025) [355] NCT05199090

*FGF21* fibroblast growth factor 21, *KLB* β-klotho, *T2D* type 2 diabetes, *MASH* metabolic dysfunction-associated steatohepatitis, *MASLD* metabolic dysfunction-associated steatotic liver disease, *DIO* diet-induced obesity, *GDF15* growth differentiation factor 15, *GFRAL* glial cell-derived neurotrophic factor family receptor α-like; -, not applicable

In contrast, most preclinical trials of GDF15 have focused on obesity (Table 3), whereas clinical studies remain limited to a few early-phase trials (Table 4). Although GDF15 analogues have shown evidence of appetite suppression and modest weight reduction in humans, their efficacy has been lower than predicted from preclinical studies, and gastrointestinal adverse events, particularly nausea and vomiting, have limited dose escalation and long-term treatment [354, 355]. Therefore, in contrast to FGF21, the clinical translation of GDF15 agonists remains limited to early-phase studies, with no ongoing registrational programs identified to date. However, similarly to FGF21, dual- and triple-agonist strategies involving GDF15 are currently being developed, but they also remain at preclinical stages [369, 370].

Notably, despite their potential complementary effects in the treatment of obesity, few studies have investigated the effects of combining FGF21 and GDF15. In a preclinical study, 25 days of treatment with recombinant FGF21 or GDF15 reduced body weight by approximately 10% and 14%, respectively. Interestingly, combined treatment resulted in an approximately 25% reduction in body weight, suggesting potential additive effects of these two pathways. The combination also improved glucose tolerance, reduced fasting insulin, and decreased hepatic TG content, indicating additional metabolic effects [371]. These findings suggest that combined targeting of FGF21 and GDF15 may represent a promising therapeutic strategy for obesity and associated metabolic disorders. However, whether these effects can be reproduced in humans and whether they offer a clinically meaningful advantage over single-pathway therapies remain unknown.

### Current challenges in clinical translation

Despite the promising preclinical results and the potential outcomes of several clinical trials, important challenges remain for the clinical translation of FGF21- and GDF15-based therapies. FGF21 analogues have demonstrated promising effects in MASLD/MASH with a safety profile, but ongoing phase III studies will determine whether these benefits can be translated into long-term clinical outcomes. Furthermore, despite robust improvements in preclinical obesity and T2D models, clinical trials have shown modest effects on weight loss and glycemic control, highlighting interspecies differences in FGF21 effects [190, 311]. For GDF15-based therapies, the main challenge is tolerability. Although GDF15 analogues markedly reduce food intake and body weight in preclinical models without major adverse events, their clinical translation has been limited by gastrointestinal adverse events, which restrict dose escalation and may contribute to the modest beneficial effects observed [354]. However, beyond obesity, GDF15-based therapies represent a potential therapeutic target in cancer-associated cachexia [338], showing promising results with the GDF15-neutralizing antibody Ponsegromab, which is currently being evaluated in phase II trials [372, 373].

Another challenge is the interpretation of elevated FGF21 and GDF15 concentrations in obesity, T2D, MASLD, and aging despite their observed beneficial effects. This phenomenon has led to the concept of FGF21 and GDF15 resistance. In the case of FGF21, this has been supported by reduced KLB expression under chronic inflammation associated with metabolic diseases and aging, limiting FGF21 responsiveness. However, this hypothesis remains controversial, as pharmacological FGF21 analogues retain metabolic efficacy despite elevated endogenous hormone concentrations. Mechanistic evidence supporting a resistant state for GDF15 is more limited. Alternatively, elevated circulating concentrations may represent adaptive responses to persistent metabolic stress rather than impaired hormone action. Consistent with this interpretation, circulating FGF21 and GDF15 levels generally decrease following interventions that improve metabolic health, including weight loss and regular exercise, whereas individuals with metabolic disease and older adults typically exhibit higher concentrations of both cytokines. Therefore, elevated FGF21 and GDF15 concentrations may be viewed as indicators of metabolic stress and compensatory adaptation rather than definitive evidence of hormone resistance. Nevertheless, distinguishing between resistance and adaptation remains critical for their potential use as disease biomarkers and the development of future therapeutic strategies.

Finally, despite the therapeutic potential of FGF21- and GDF15-based strategies, substantial inter-individual variability in treatment response persists, and predictors of therapeutic efficacy are currently lacking. Therefore, identifying patient populations most likely to benefit from these interventions will be essential for the implementation of precision medicine approaches. Longitudinal clinical studies could also clarify whether the combined assessment of FGF21 and GDF15 provides information beyond either mediator alone and whether specific FGF21-GDF15 patterns could identify distinct metabolic phenotypes or predict treatment responses. In addition, studies targeting both mediators could determine whether combined FGF21- and GDF15-based strategies can enhance or complement their individual therapeutic effects.

## Remaining unresolved questions

Despite extensive research on FGF21 and GDF15, several questions remain unresolved. Here we summarize key unresolved questions that need further investigation. Regarding GDF15, its known receptor, GFRAL, is primarily expressed in the brainstem. However, GDF15 exerts systemic peripheral effects, making the identification of possible additional peripheral receptors essential for understanding its peripheral actions. Moreover, the physiological roles of FGF21 and GDF15 under normal conditions are not fully understood, and although FGF21 has been traditionally more associated with energy expenditure and GDF15 with energy intake, emerging evidence indicates that both hormones can influence aspects of each other. The extent to which they contribute to integrated energy balance remains under investigation. Furthermore, although multiple tissues can produce these cytokines, the relative contributions of different organs across diseases and diverse metabolic contexts, including both physiological and pathological challenges, remain to be clarified, as do their potential interactions with other hormonal circuits. Tissue-specific and inducible loss-of-function animal models targeting metabolically relevant organs and the CNS will be important to analyse the individual and combined functions of FGF21 and GDF15, and to determine whether their effects are complementary, overlapping, additive or synergistic, as well as to establish the directionality and tissue specificity of their potential cross-regulation. Complementary cellular loss-of-function models in relevant cell types could further help dissect the intracellular pathways mediating their interaction. Moreover, combined or sequential manipulation of both pathways could further establish whether the effects of one mediator are maintained, attenuated, or compensated in the absence of the other. In parallel, integrative multi-omics approaches and single-cell or spatial analyses could help identify shared and mediator-specific molecular signatures and determine whether FGF21 and GDF15 converge on common signaling pathways or activate distinct tissue-specific programs.

Additional questions include the differential effects of acute versus chronic stress, and of physiological versus pathological conditions, as well as the possible sex- and age-dependent differences in response to FGF21 and GDF15 treatments. These variables may influence the magnitude, timing, and tissue sources of FGF21 and GDF15 responses, and may be particularly relevant for determining whether apparently divergent findings across experimental models reflect biological differences or differences in the experimental context. Importantly, mechanistic findings from experimental models will need to be validated in humans.

Finally, although both FGF21 and GDF15 are induced by similar cellular stressors and share overlapping protective effects, their distinct yet complementary biological functions and mechanisms of action suggest a coordinated endocrine stress response to restore metabolic balance and protect against tissue injury. Whether they act cooperatively within this endocrine stress-response or instead represent parallel but independent stress signals remain unclear. Emerging evidence suggests that they may form part of a broader endocrine stress-response network; however, the mechanisms, physiological relevance, and potential context-dependence of their interaction, including whether it involves direct cross-regulation, and the potential redundancy or compensatory interactions between them, remain largely unresolved.

## Conclusions

Overall, by integrating current evidence from FGF21 and GDF15, which have traditionally been investigated independently, this review highlights the possibility that they may represent interconnected components of the endocrine response to cellular stress and tissue dysfunction underlying metabolic diseases and aging. Their elevation in obesity, T2D, MASLD and aging has positioned them as promising biomarkers for disease progression and response to metabolic interventions, while they also actively participate in adaptive responses aimed at maintaining systemic homeostasis. Although the extent of their possible interaction remains incompletely understood, available evidence supports complementary, and potentially synergistic, roles of FGF21 and GDF15 in preserving systemic health.

This review highlights that the biological meaning of increased FGF21 and GDF15 concentrations is unlikely to be uniform across physiological and pathological states. Their effects may vary according to the tissue involved, the nature and duration of the underlying stress, and the capacity of the organisms to respond. Thus, their elevation may initially reflect an adaptive response aimed at preserving metabolic homeostasis, whereas persistent elevation in chronic disease or aging may indicate sustained tissue stress and reflect an insufficient compensatory response to progressive tissue dysfunction. Interestingly, our integration of the available evidence not only highlights complementary and partially overlapping functions of FGF21 and GDF15 in the regulation of energy balance and cellular stress but also identifies emerging evidence of cross-regulation between them, which appears to be highly dependent on the tissue and context.

A better understanding of the coordinated regulation and actions of FGF21 and GDF15 may therefore open new opportunities for the development of combined therapeutic strategies aimed at reducing body weight and mitigating inflammation, metabolic dysfunction, and age-related deterioration.

## Data Availability

Not applicable.
